# Multi-dimensional identification and quality evaluation of *Paris polyphylla* cultivated populations using morphological, molecular, cytological and metabolic traits

**DOI:** 10.3389/fpls.2026.1878411

**Published:** 2026-08-13

**Authors:** Jinhui Jiang, Yirong Qin, Zhongquan Yu, Junwen Chen, Jianjun Wang, Shengchao Yang, Yu Liu, Tao Liu

**Affiliations:** 1 College of Agronomy and Biotechnology, Yunnan Agricultural University, Kunming, China; 2 National-Local Joint Engineering Research Center on Germplasm Innovation & Utilization of Chinese Medicinal Materials in Southwest China, Yunnan Agricultural University, Kunming, Yunnan, China; 3 College of Biological and Agricultural Sciences, Honghe University, Mengzi, China; 4 Good Clinical Practice (GCP) Department, Shenzhen Traditional Chinese Medicine Hospital, The Fourth Clinical Medical College of Guangzhou University of Chinese Medicine, Shenzhen, China

**Keywords:** cultivated populations, DNA barcode, genome size, karyotype, *Paris polyphylla*, phenotypic variation

## Abstract

**Introduction:**

*Paris polyphylla* is an endangered medicinal herb valued for its steroidal saponins, yet the phenotypic stability, genetic diversity, and quality differentiation of cultivated populations remain insufficiently understood.

**Methods:**

In this study, 28 representative populations from Yunnan Province were evaluated under common-garden conditions using morphological, molecular, cytogenetic, genomic and phytochemical analyses.

**Results:**

The populations showed generally synchronized phenological development but differed significantly in several agronomic traits. DNA barcoding based on ITS and *trnL–trnF* sequences achieved high amplification and sequencing success. Among the representative sequences examined, ITS exhibited greater haplotype richness and sequence variation than *trnL–trnF*. However, because each population was represented by only one consensus sequence, within-population variation and a conventional DNA barcode gap could not be evaluated. Flow cytometry and chromosome counting confirmed that all populations were diploid, although substantial variation in genome size and karyotype characteristics was observed. HPLC analysis further revealed marked differences in steroidal saponin composition and content among populations.Correlation analyses linked saponin variation to multiple agronomic traits, karyotypic features, and genome size. Exploratory partial least squares path modeling showed that temperature was positively associated with plant growth, whereas plant growth was negatively associated with the composite saponin profile. The model explained a limited proportion of the variation in the endogenous constructs, and the identified paths were interpreted as statistical associations rather than causal effects.

**Discussion:**

Overall, this study clarifies the genetic and ecological drivers of saponin variation in cultivated *P. polyphylla*, providing an integrated framework for germplasm identification, quality evaluation, and sustainable utilization of cultivated resources.

## Graphical Abstract

**Figure:**
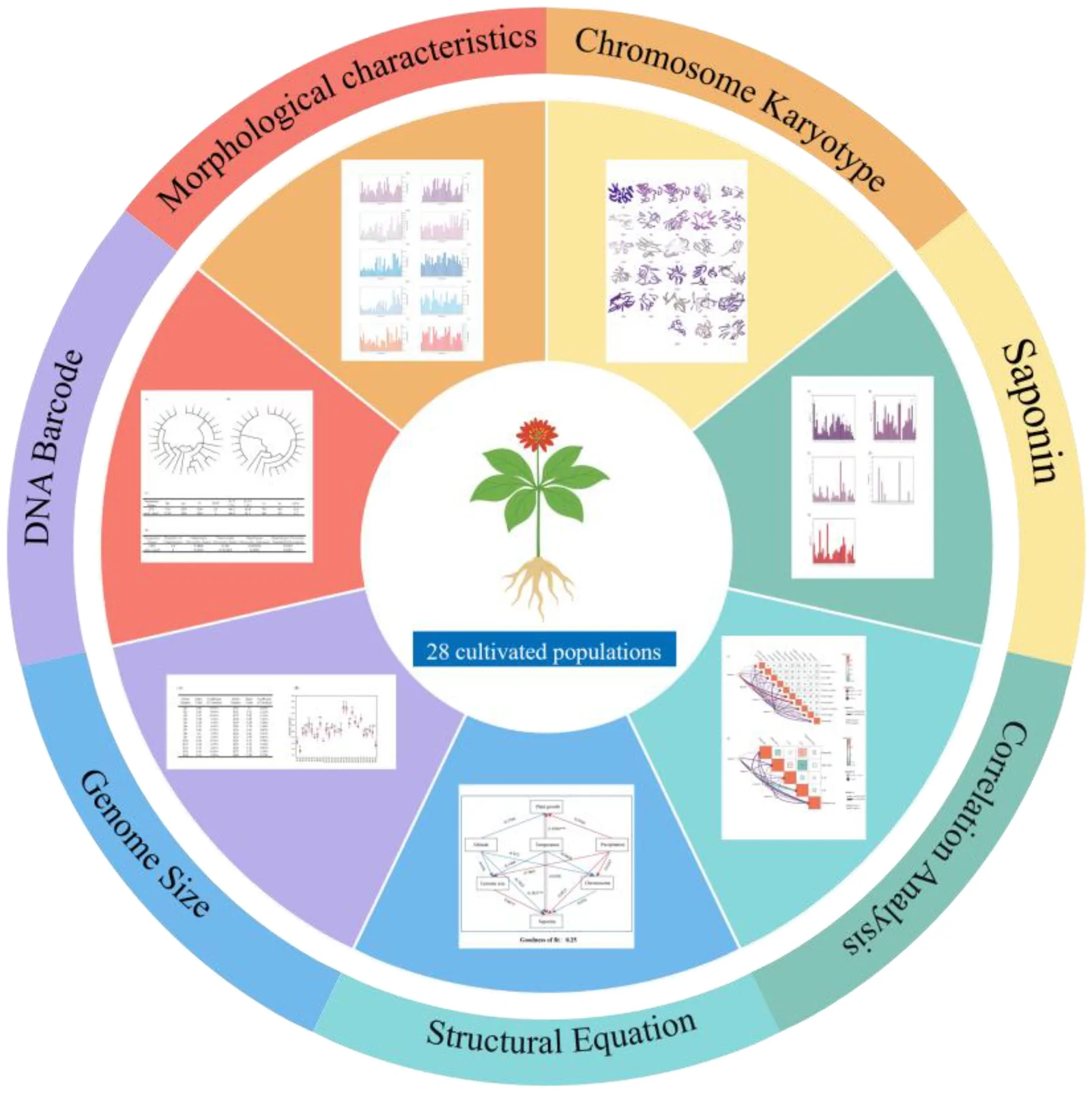

## 1 Introduction

*Paris polyphylla* var. *yunnanensis*, a member of the family Melanthiaceae according to modern angiosperm classification systems, is mainly distributed in southwestern China, including Yunnan, Sichuan, and Guizhou, where it occurs in evergreen broad-leaved forests at elevations of 1,400–3,100 m (Ding et al., 2021; Ji et al., 2006; The Angiosperm Phylogeny Group, 2016). Its rhizomes are widely used in traditional Chinese medicine for the treatment of traumatic injuries, mastitis, bronchitis, and bone fractures (Cunningham et al., 2018; Qiang et al., 2020; Wu et al., 2012). The major secondary metabolites of *P. polyphylla* include steroidal saponins, phytosterols, flavonoids, polysaccharides, and amino acids, among which steroidal saponins (such as polyphyllin I, II, and VII) account for approximately 80% of the total active constituents (Liu et al., 2019; Wang et al., 2017).These saponins exhibit diverse pharmacological activities, including anticancer, immunomodulatory, anti-inflammatory, and antidiabetic effects, and serve as important precursors for steroidal drug synthesis (Li et al., 2023). The annual market demand for *P. polyphylla* rhizomes in the pharmaceutical industry is estimated at around 3,000 tons (Cunningham et al., 2018). However, due to its long growth cycle, overharvesting, and habitat destruction, wild populations have been largely depleted (Yang et al., 2018b). Consequently, cultivated *P. polyphylla* has become the primary source of medicinal material. Yet, confusion regarding germplasm authenticity, complex provenance of cultivated varieties, and the absence of effective identification methods have hindered systematic germplasm management and the fixation of elite genotypes. A comprehensive morphological and molecular characterization of cultivated populations from different regions would help elucidate genetic divergence and ecological adaptation, providing a scientific foundation for germplasm evaluation, breeding of superior lines, and standardization of medicinal quality.

DNA barcoding utilizes conserved genomic regions for rapid and accurate species identification (Yang et al., 2018a; Zhang et al., 2023). Over the past two decades, it has been validated as a robust molecular tool across diverse biological fields. For instance, the cytochrome c oxidase subunit I (COI) gene has become the standard marker for animal species identification (Fernandes et al., 2021). In plants, regions such as ITS2, *psbA–trnH*, *rbcL*, and *matK* are considered promising barcode candidates. In 2014, Chen et al. (Chen et al., 2014) established a DNA barcode database integrating ITS2 and *psbA–trnH* sequences, which provided a species authentication platform covering over 95% of herbal materials listed in the Chinese, Japanese, European, and U.S. pharmacopoeias. The application of DNA barcoding has since expanded considerably, particularly in the authentication of medicinal plant materials (Chen et al., 2023). Fushimi et al. (Fushimi et al., 1996) extracted DNA from fresh plant tissues and processed herbal samples of *Panax* species and demonstrated that nucleotide sequence variation can be used as genetic markers to distinguish between *Panax ginseng* and related herbs. Wen et al. (Wen and Zimmer, 1996) analyzed the ITS sequences of twelve *Panax* species and found that *P. quinquefolius* shares closer phylogenetic relationships with East Asian taxa such as *P. ginseng*, *P. japonicus*, and *P. notoginseng*. Similarly, Ngan et al (Ngan et al., 1999)successfully differentiated six medicinal *Panax* species and two common adulterants (*Mirabilis jalapa* and *Phytolacca americana*) using ITS sequence analysis. In this study, we compared sequence variation and phylogenetic resolution of ITS and *trnL–trnF* among representative sequences from cultivated populations to evaluate their potential as molecular markers for germplasm differentiation.

In higher plants, karyotype analysis is widely used to compare chromosome morphology and number among species or varieties, providing key insights into plant origin, evolution, molecular phylogeny, and biogeography. It also facilitates species classification and aids in breeding programs by identifying cytogenetic parameters and confirming alien chromosome additions (Farshadfar, 2020; Guerra, 2008; Sun WenGuang et al., 2019). Karyotype characterization typically includes chromosome number, absolute and relative length, centromere position, distribution of heterochromatin regions, rDNA loci, and the degree of asymmetry (Maoxue and Ruiyang, 1985; She and Jiang, 2015). Chromosome number and karyotype are generally stable within a species or variety; variation in these parameters among populations can lead to reproductive isolation, thereby maintaining species integrity and stability. The arm ratio (the ratio of the longest to the shortest chromosome) represents a fundamental karyotypic feature of a genotype. By combining the arm ratio and the proportion of chromosomes with arm ratios less than 2:1, Stebbins (Stebbins, 1971)proposed a karyotype classification system widely used in plant cytogenetic studies.

Flow cytometry (FCM) is a rapid and reliable method for estimating nuclear genome size and has been successfully applied in ploidy identification, cell cycle analysis, and species discrimination, including the detection of hybrids, rare cytotypes, and aneuploids (Francis et al., 2008; Hanušová et al., 2014; Vit et al., 2014; Zhang et al., 2019). This technique has been extensively employed in genetic diversity analysis, breeding, reproductive ecology, evolution, and plant systematics (Bilinski et al., 2018; Galbraith, 2004; Sharma et al., 2019; Španiel et al., 2019). FCM offers several advantages such as simplicity, high accuracy, non-destructive sampling, and suitability for large-scale screening using minimal tissue quantities (Dolezel, 1997).

As a traditional medicinal plant officially listed in the Chinese Pharmacopoeia, *P. polyphylla* exhibits remarkable anticancer activity and possesses broad pharmacological and functional potential. Given the depletion of wild resources and challenges in artificial cultivation and breeding, a comprehensive evaluation of existing cultivated populations is urgently needed to identify superior germplasms, promote standardized cultivation, and improve medicinal quality. The present study aimed to investigate the synergistic effects of ecological and genetic factors on saponin accumulation by integrating plant morphology, molecular biology, cytogenetics, and metabolite profiling. Our goal was to establish an integrative evaluation system for the identification of elite germplasms and quality assessment of *P. polyphylla*, providing a scientific basis for the breeding of superior cultivars and quality regulation in the herbal medicine industry.

## 2 Materials and methods

### 2.1 Plant materials

A total of 28 cultivated populations of *Paris polyphylla* var. *yunnanensis* were collected from different cultivation sites across Yunnan Province, China. Five healthy plants of similar age were selected from each population. Before transplantation, phenological observations and morphological measurements were conducted at their original cultivation sites. At the same time, the geographical coordinates and altitude of each cultivation site were recorded during the field survey. Mean annual temperature and annual precipitation for the period from 2023 to 2024 were obtained from the China Meteorological Administration (CMA) according to the geographic coordinates of each cultivation site. These climatic variables were used as source-site environmental variables in the subsequent analyses.

After completion of the field investigation, the selected plants were transplanted to the experimental base of Yunnan Agricultural University (25.14° N, 102.76° E), where they were maintained under uniform cultivation and management conditions. The transplanted materials were subsequently used for DNA barcoding, flow-cytometric genome-size estimation, chromosome karyotype analysis, and steroidal saponin determination.

### 2.2 Experimental design

Phenological characteristics and ten quantitative morphological traits were investigated in the original cultivation sites using five biological replicates from each population. Following completion of the field survey, the selected plants were transplanted to the common experimental garden at Yunnan Agricultural University and cultivated under identical management conditions.

After the plants had established under the common-garden conditions, leaf samples were collected for DNA extraction, young leaves were sampled for flow-cytometric genome-size estimation, actively growing rhizome tips were harvested for chromosome karyotype analysis, and rhizome tissues were collected for steroidal saponin quantification. All laboratory analyses were performed using materials grown under the same cultivation conditions to minimize environmental variation during sampling.

Because morphological traits were measured before transplantation, the observed phenotypic variation reflects the combined effects of population genetic differentiation and local cultivation environments. In contrast, the molecular, cytogenetic, genome-size, and phytochemical analyses were conducted using plants maintained under common-garden conditions to reduce variation associated with sampling conditions.

### 2.3 Morphological characterization

Morphological characterization was conducted at the original cultivation sites before transplantation. During the field investigation, phenological characteristics, including sprouting, flowering, fruit dehiscence, and senescence, were recorded. Ten quantitative agronomic traits, including leaf number, sepal number, stem diameter, leaf length, stamen number, leaf width, petiole length, peduncle length, plant height, and crown width, were measured using standardized methods. Environmental information was compiled for each original cultivation site. Latitude, longitude, and altitude were recorded during the field survey, whereas mean annual temperature and annual precipitation for the period from 2023 to 2024 were obtained from the China Meteorological Administration based on the geographic coordinates of each cultivation site.

### 2.4 DNA extraction

Genomic DNA was extracted from dried leaf tissues (~30 mg) using a Plant Genomic DNA Extraction Kit (Zhuangmeng Biotech, China) following the manufacturer’s instructions. DNA quality and quantity were verified using 1% agarose gel electrophoresis and spectrophotometric analysis, and the extracts were stored at −20 °C for subsequent PCR amplification.

### 2.5 PCR amplification and sequencing

PCR amplification was carried out in a 30 μL reaction containing 1.5 μL DNA, 15 μL 2×Taq PCR StarMix, 0.6 μL each of forward and reverse primers, and 12.3 μL ddH_2_O. The amplification program consisted of an initial denaturation at 94 °C for 2 min, followed by 25 cycles of 94 °C for 30 s, 55 °C for 30 s, and 72 °C for 1 min, with a final extension at 72 °C for 5 min. PCR products were verified by agarose gel electrophoresis and purified prior to sequencing. The ITS (ITS1, ITS4) (White et al., 1990) and *trnL–trnF* (Taberlet et al., 1991)regions were amplified using universal primers:

ITS1: 5′-TCCGTAGGTGAACCTGCGG-3′; ITS4: 5′-TCCTCCGCTTATTGATATGC-3′.

*trnL–trnF* forward: 5′-ATTTGAACTGGTGACACGAG-3′; reverse: 5′-CGAAATCGGTAGACGCTACG-3′.

PCR products were sequenced bidirectionally by Qingke Biotechnology Co., Ltd. (Beijing, China), with three technical replicates per sample to ensure accuracy. Although multiple DNA extracts were initially screened, only one high-quality representative sequence per cultivated population was retained for each marker and used in the subsequent sequence analyses.

### 2.6 Flow cytometric analysis

Nuclear genome size was estimated by flow cytometry at the Molecular Biology Experiment Center of the Germplasm Bank of Wild Species in Southwest China, Kunming Institute of Botany, Chinese Academy of Sciences. *Triticum aestivum* L. cv. Chinese Spring was used as the internal reference standard because its reference genome has been comprehensively assembled and extensively characterized (CONSORTIUM et al., 2018). The published 1C reference genome size of 14.5 Gbp was used as the calibration value for genome-size estimation.

Fresh young leaves (approximately 0.5 cm²) from each sample and the Chinese Spring reference were chopped separately in 0.8 mL of pre-cooled MGb buffer containing 45 mM MgCl_2_·6H_2_O, 20 mM MOPS, 30 mM sodium citrate, 1% PVP-40, 0.2% Triton X-100, 10 mM Na_2_EDTA, and 20 μL mL^-1^ β-mercaptoethanol (pH 7.5). After incubation on ice for 10 min, the nuclear suspension was filtered through a 40-μm nylon mesh, stained with propidium iodide (50 μg mL^-1^) and RNase A (50 μg mL^-1^), and incubated in darkness for 30–60 min following the standard protocol for plant nuclear DNA estimation (Dolezel, 2005; Doležel et al., 2007).

The stained nuclei from the sample and the Chinese Spring reference were mixed immediately before measurement and analyzed simultaneously using a BD FACSCalibur flow cytometer (BD Biosciences, USA) equipped with a 488-nm argon laser. Histograms were analyzed using ModFit LT 3.0 software. At least 10,000 nuclei were recorded for each measurement, and only histograms with a coefficient of variation (CV) below 5% were accepted for further analysis.

Genome size was calculated according to the following equation:


$$Sample\;1C\;genome\;size\;\left(Gbp\right)=14.5\;Gbp\times \frac{Mean\;fluorescence\;intensity\;of\;sample\;G_{0}/G_{1}\;peak}{Mean\;fluorescence\;intensity\;of\;Chinese\;Spring\;G_{0}/G_{1}\;peak}$$

Both the sample and reference fluorescence peaks corresponded to G_0_/G_1_ nuclei (2C DNA content). Because both peaks were measured at the same cell-cycle stage, the fluorescence ratio represents the relative DNA-content ratio between the sample and the reference. The calculated ratio was normalized to the published 1C genome size of Chinese Spring (14.5 Gbp); therefore, all genome-size values reported in this study represent 1C genome sizes unless otherwise stated. The experimental procedure generally followed the flow-cytometric protocol for plant genome-size estimation described by Doležel and Bartoš (Dolezel, 2005).

### 2.7 Chromosome karyotype analysis

Actively growing rhizome tips (2–3 cm) were collected from each of three individuals per population between July and October (10:00–11:00 a.m.). Rhizome tips were pretreated with 8-hydroxyquinoline for 5–8 h, fixed in Carnoy’s solution (ethanol:acetic acid = 3:1) for 24 h, and stored at 4 °C. Fixed samples were hydrolyzed in 1 M HCl at 60 °C for 5–8 min, rinsed with distilled water, and preserved in 75% ethanol at 4 °C. Rhizome tips were stained with carbol fuchsin, gently squashed under a coverslip, and observed under a light microscope. Well-spread metaphase cells with clear chromosomes were photographed for further karyotype analysis.

### 2.8 Determination of steroidal saponins

Three individuals were selected per population. Rhizome samples were cut from the middle portion, dried at 45 °C to a constant weight, ground into fine powder (100 mesh), and analyzed according to the methods described in the *Chinese Pharmacopoeia* (2020 edition). The rhizome is the medicinal organ specified in the Chinese Pharmacopoeia. Quantification of major saponins (polyphyllin I, II, VI, VII, D, and H) was performed using an Agilent ZORBAX SB-Aq column (250 mm × 4.6 mm). The mobile phase consisted of acetonitrile (A) and water (B) with a gradient of 0–40 min (30:70 → 60:40), 40–41 min (60:40 → 80:20), and 41–44 min (80:20). The detection wavelength was set at 203 nm, the column temperature at 30 °C, the flow rate at 1.0 mL min^-1^, and the injection volume at 20 μL. Representative chromatograms of the mixed reference standards and a cultivated-population sample were included in the **Supplementary Figure S1** to verify peak identification. The mixed reference-standard solution was reinjected three times, and the results showed stable retention times and peak responses, providing preliminary evidence of injection precision and chromatographic stability. However, because the quantitative analysis was completed previously using a routine analytical service, the original validation records required to reconstruct calibration curves, linear ranges, limits of detection (LOD), limits of quantification (LOQ), and spike-recovery data were unavailable. Therefore, these validation parameters could not be retrospectively reported in the present study.

### 2.9 Data analysis

Morphological traits, genome size, and saponin contents were analyzed using SPSS 26.0 (IBM Corp., Armonk, NY, USA). Data are presented as the mean ± standard deviation. The normality of residuals and homogeneity of variances were examined before inferential analysis. Differences among cultivated populations were evaluated using one-way analysis of variance. When the overall ANOVA was significant, Tukey’s honestly significant difference test was applied for *post hoc* multiple comparisons. The F statistic, numerator and denominator degrees of freedom, and exact P value were reported. Different lowercase letters in figures and tables indicate significant differences among populations at P< 0.05. Chromosome measurements and karyotype analyses were performed using MATO software (Liu et al., 2023).

Pearson correlation analysis was performed to evaluate linear relationships among quantitative variables. Pearson’s correlation coefficient was selected because the variables approximately satisfied the assumptions of normality and linearity after preliminary examination. Mantel tests were subsequently performed to evaluate the correlations between different groups of variables.

The ITS and trnL–trnF sequences were aligned and analyzed using MEGA 7.0 (Kumar et al., 2016). The original multiple-sequence alignments were inspected to identify nucleotide substitutions and alignment gaps. Alignment gaps were interpreted cautiously as putative insertion/deletion events. Sequence-polymorphism and genetic-diversity indices, including alignment length, polymorphic sites (S), singleton variable sites, parsimony-informative sites, number of haplotypes (h), haplotype diversity (Hd), and nucleotide diversity (π), were calculated using DnaSP (Librado and Rozas, 2009). Phylogenetic relationships among the 28 cultivated populations were reconstructed using the Neighbor-Joining method based on p-distances (Saitou and Nei, 1987; Kumar et al., 2016). Both transitional and transversional substitutions were included, and uniform evolutionary rates among sites were assumed. The original multiple-sequence alignments were first inspected to identify nucleotide substitutions and alignment gaps. Alignment gaps were interpreted cautiously as putative insertion/deletion events. For phylogenetic-tree reconstruction, sites containing gaps or missing data were subsequently excluded using the complete-deletion option. The reliability of the resulting tree topology was evaluated using 1,000 bootstrap replicates (Felsenstein, 1985).

Partial least squares path modeling was used to explore associations among source-site altitude, mean annual temperature, mean annual precipitation, karyotypic characteristics, genome size, plant-growth traits, and saponin profiles. PLS-PM was selected because it allows the simultaneous evaluation of relationships among multiple observed and latent variables, does not require strict multivariate normality, and is suitable for exploratory analyses involving relatively small samples (Hair et al., 2019). Model performance was evaluated using the global goodness-of-fit index and the coefficients of determination of the endogenous constructs. Because the current model included repeated measurements nested within 28 populations and bootstrap-based validation and a reliable Q² index were unavailable, the PLS-PM was regarded as exploratory. Because standardized measurements of soil physicochemical properties, canopy shading, light availability, irrigation, fertilization, and cultivation management were unavailable for all 28 cultivation sites, these environmental variables were not incorporated into the current PLS-PM. Path coefficients were therefore interpreted as statistical associations rather than evidence of causality. The significance of path coefficients was assessed using the linear-model significance tests implemented in the plspm package. Because bootstrap confidence intervals were not calculated, the PLS-PM was interpreted as an exploratory model. Figures were generated using Origin 2021 (OriginLab Corporation, Northampton, MA, USA).

## 3 Results

### 3.1 Morphological variation among cultivated populations of *Paris polyphylla* var. *yunnanensis*

**Figure 1:**
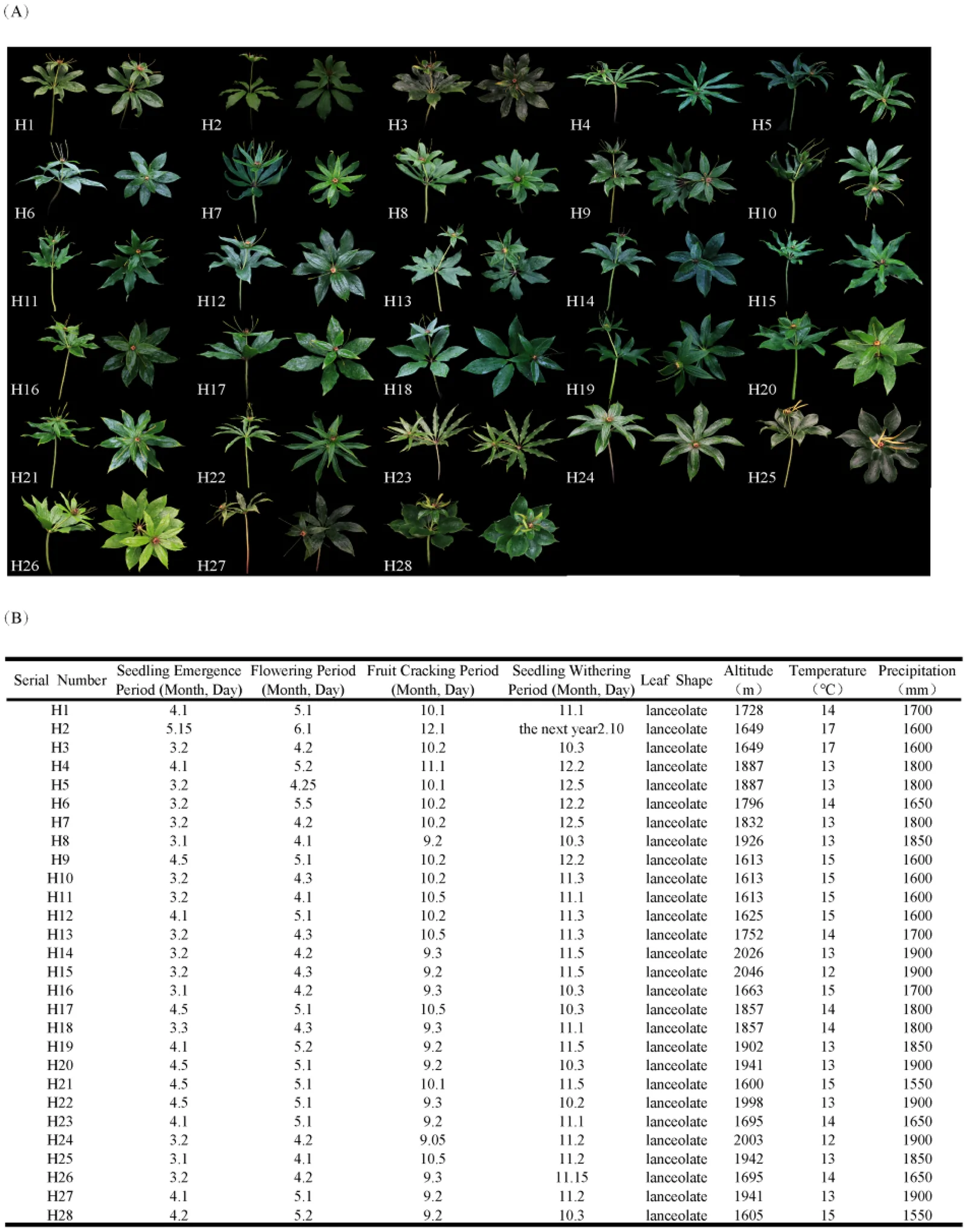
Profile of 28 cultivated populations of *Paris polyphylla*. **(A)** Foliar morphology of the 28 cultivated populations; **(B)** Phenological stages and environmental conditions of the 28 cultivated populations of *Paris polyphylla*.

Between 2023 and 2024, 28 cultivated populations of *P. polyphylla* were transplanted to the experimental site at Yunnan Agricultural University and monitored for phenology and leaf morphology (**Figure 1**). The results indicated that these 28 populations exhibited a relatively synchronized emergence period, from early March to mid-May. The earliest population, H8, emerged on 1 March, and the latest, H2, on 15 May. Flowering occurred approximately one month after emergence, with H8 flowering on 1 April and H2 on 1 June. Fruit dehiscence spanned a broader range, from early September (e.g., H8, 2 September) until early December (H2, 1 December). Most plants entered senescence (seedling wilting) between October and December; notably, population H2 delayed senescence until 10 February of the following year, suggesting distinctive physiological traits or micro-environmental influences. All 28 populations exhibited lanceolate leaf morphology and occupied collection altitudes ranging from 1,600 to 2,046 m and mean annual temperatures of 12–17 °C.

**Figure 2:**
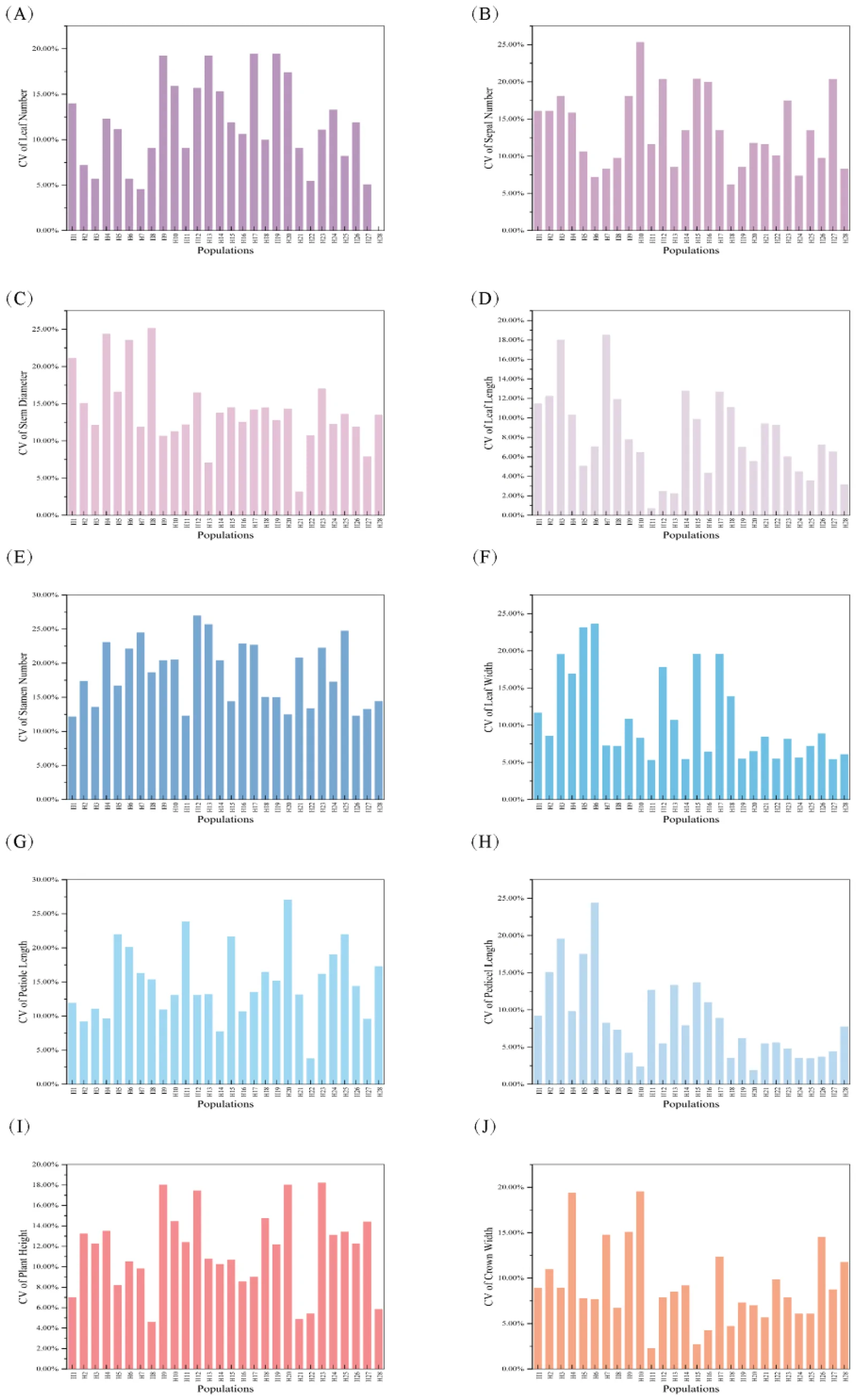
Histogram of the comprehensive coefficient of variation (CV) for ten traits across different cultivated populations of *Paris polyphylla* var. yunnanensis.CV of **(A)** leaf number; **(B)** sepal number; **(C)** stem diameter; **(D)** leaf length; **(E)** stamen number; **(F)** leaf width; **(G)** petiole length; **(H)** pedicel length; **(I)** plant height; **(J)** crown width.

To assess phenotypic stability and variation across populations, coefficients of variation (CV) were calculated for ten agronomic traits (leaf number, sepal number, stem diameter, leaf length, stamen number, leaf width, petiole length, peduncle length, plant height, crown width) (**Figure 2**). The CV ranges were: leaf number 0–19.98%, sepal number 5.82–25.35%, stemdiameter 3.19–25.20%, leaf length 0.73–18.52%, stamen number 12.16–27.00%, leaf width 5.35–23.65%, petiole length 3.79–27.05%, peduncle length 1.92–24.41%, plant height 2.28–18.24%, crown width 2.71–19.42%. Petiole length exhibited the greatest variation (3.79–27.05%), indicating that this trait was the least stable among individuals within populations. In contrast, leaf length exhibited the lowest minimum CV (0.74%, in population H11), indicating high trait consistency within that population. Moreover, H11 had the lowest CV values among three traits (leaf length, leaf width, plant height), suggesting that multiple traits in H11 were consistently stable. These data indicate that genetic stability differs fundamentally among morphological traits. One-way ANOVA revealed significant differences among the 28 cultivated populations for leaf number (F_(27,112)_ = 3.502, P< 0.001), sepal number (F_(27,112)_ = 4.137, P< 0.001), stem diameter (F_(27,112)_ = 3.961, P< 0.001), leaf length (F_(27,112)_ = 13.967, P< 0.001), leaf width (F_(27,112)_ = 7.544, P< 0.001), petiole length (F_(27,112)_ = 4.757, P< 0.001), peduncle length (F_(27,112)_ = 15.894, P< 0.001), plant height (F_(27,112)_ = 4.544, P< 0.001), and crown width (F_(27,112)_ = 14.412, P< 0.001). In contrast, stamen number did not differ significantly among populations (F_(27,112)_ = 1.321, P = 0.158). Detailed Tukey HSD groupings are provided in **Supplementary Table 1**.

### 3.2 DNA-barcoding discrimination among cultivated populations

**Figure 3:**
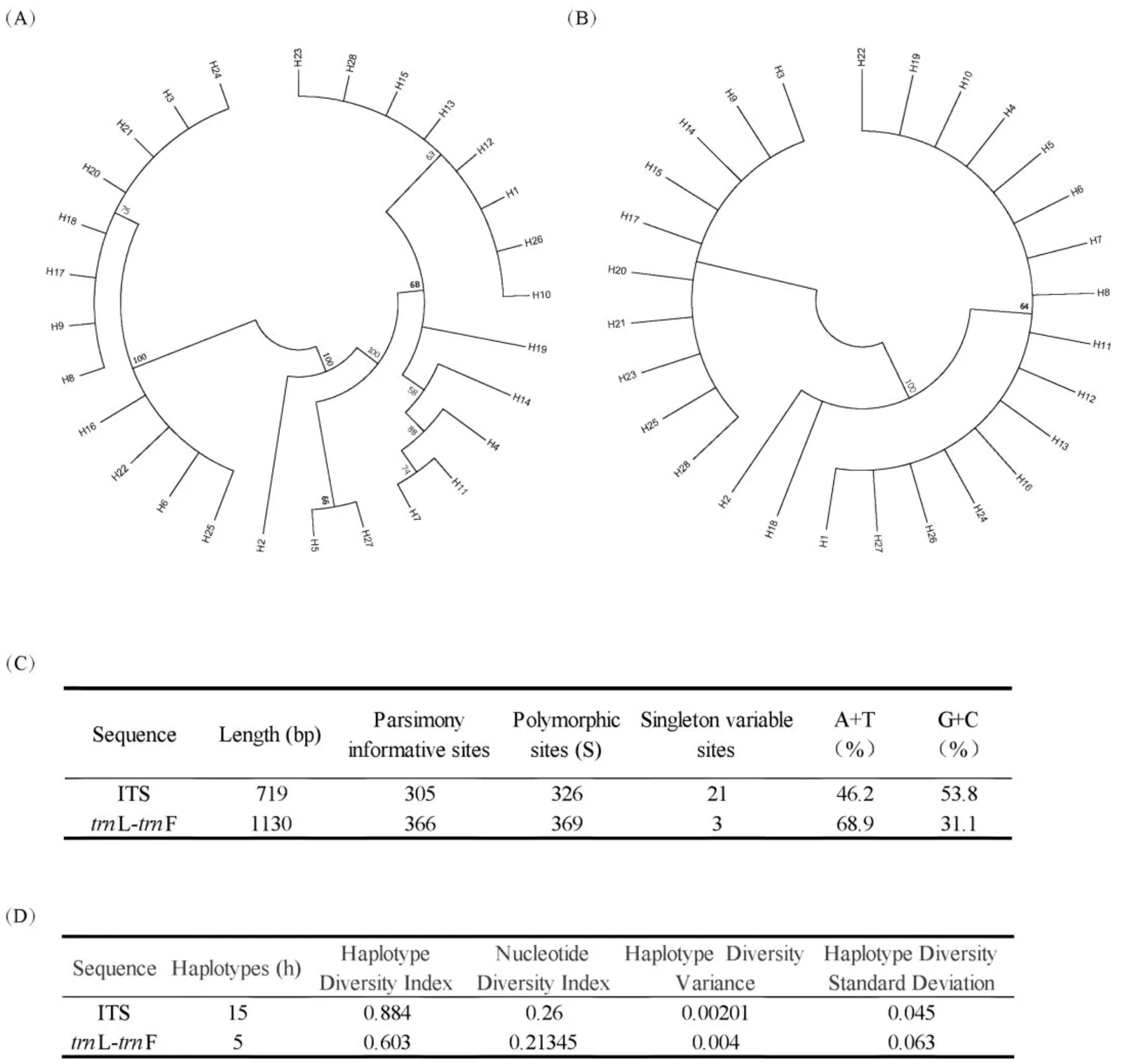
DNA-barcoding analysis of 28 cultivated populations of Paris polyphylla. **(A)** Neighbor-joining phylogenetic tree based on ITS sequences. **(B)** Neighbor-joining phylogenetic tree based on trnL–trnF sequences. The trees were reconstructed in MEGA 7.0 using p-distances. Both transitional and transversional substitutions were included, uniform evolutionary rates among sites were assumed, and sites containing gaps or missing data were excluded using the complete-deletion option. Branch support was evaluated using 1,000 bootstrap replicates, and bootstrap values are shown adjacent to the corresponding branches. **(C)** Sequence characteristics of ITS and trnL–trnF, including alignment length, parsimony-informative sites, polymorphic sites (S), singleton variable sites, and nucleotide composition. **(D)** Genetic-diversity indices of the two barcode regions. h, number of haplotypes; Hd, haplotype diversity; π, nucleotide diversity; SD, standard deviation. Each cultivated population was represented by one consensus sequence for each marker.

Leaf samples were collected, silica-dried, and DNA extracted; OD values were measured and three high-quality DNA extracts per population were initially screened for PCR amplification. After bidirectional sequencing, sequence assembly, and quality control, one consensus sequence per population was retained as the representative sequence for each marker and used in subsequent analyses. Two molecular markers, nuclear ITS (ITS1/ITS4) and chloroplast *trnL–trnF*, were amplified and sequenced. Electrophoresis showed clear bands; sequencing success was 98.58% for ITS and 99.29% for *trnL–trnF*. Sequence alignment revealed an ITS alignment length of 719 bp and a trnL–trnF alignment length of 1,130 bp, including alignment gaps (**Figure 3C**). The trnL–trnF region exhibited a high A+T content of 68.9%, whereas ITS showed a higher G+C content of 53.8%. Sequence-polymorphism analysis identified 326 polymorphic sites in ITS, including 21 singleton variable sites and 305 parsimony-informative sites. In comparison, trnL–trnF contained 369 polymorphic sites, including 3 singleton variable sites and 366 parsimony-informative sites. Nucleotide substitutions and alignment gaps were observed in both datasets. Because alignment gaps may correspond to insertion/deletion events, these variation types were described separately and were not used to infer that either region was inherently more conserved than the other. Representative sequence alignments are provided in **Supplementary Figures S2** and **S3**.

Genetic diversity analyses (**Figure 3D**) showed that the ITS region harbored 15 haplotypes (haplotype diversity Hd = 0.884; nucleotide diversity π = 0.260; variance = 0.00201; SD = 0.045). In contrast, *trnL–trnF* contained 5 haplotypes (Hd = 0.603; π = 0.213; variance = 0.00400; SD = 0.063). Thus, ITS exhibited higher haplotype diversity and greater haplotype richness than trnL–trnF in the present dataset. These differences indicate distinct patterns of genetic variation and marker performance between the two loci rather than direct evidence that one region is inherently more conserved than the other. Bootstrap values ≥90% were regarded as relatively strong support, values of 70–89% as moderate support, and values below 70% as weak support. Neighbor-Joining phylogenetic trees based on p-distances were constructed for the ITS and trnL–trnF datasets using MEGA 7.0, and branch support was evaluated using 1,000 bootstrap replicates. The Neighbor-Joining tree based on ITS sequences contained several moderately to strongly supported clusters (**Figure 3A**). For example, populations H2 and H25 clustered together with 100% bootstrap support, whereas H3, H17, H18, and H21 formed a terminal cluster with 77% bootstrap support. These results indicated that ITS resolved several groupings among the representative population sequences, although the relationships associated with low-bootstrap internal nodes should be interpreted cautiously. In the Neighbor-Joining tree based on trnL–trnF sequences (**Figure 3B**), H18, H28, H3, and H25 formed a strongly supported cluster with 100% bootstrap support, while H2 was associated with this cluster with 89% bootstrap support. However, several internal branches showed relatively low bootstrap support, indicating uncertainty in some of the inferred relationships among the representative sequences.

### 3.3 Flow cytometric determination of genome size

**Figure 4:**
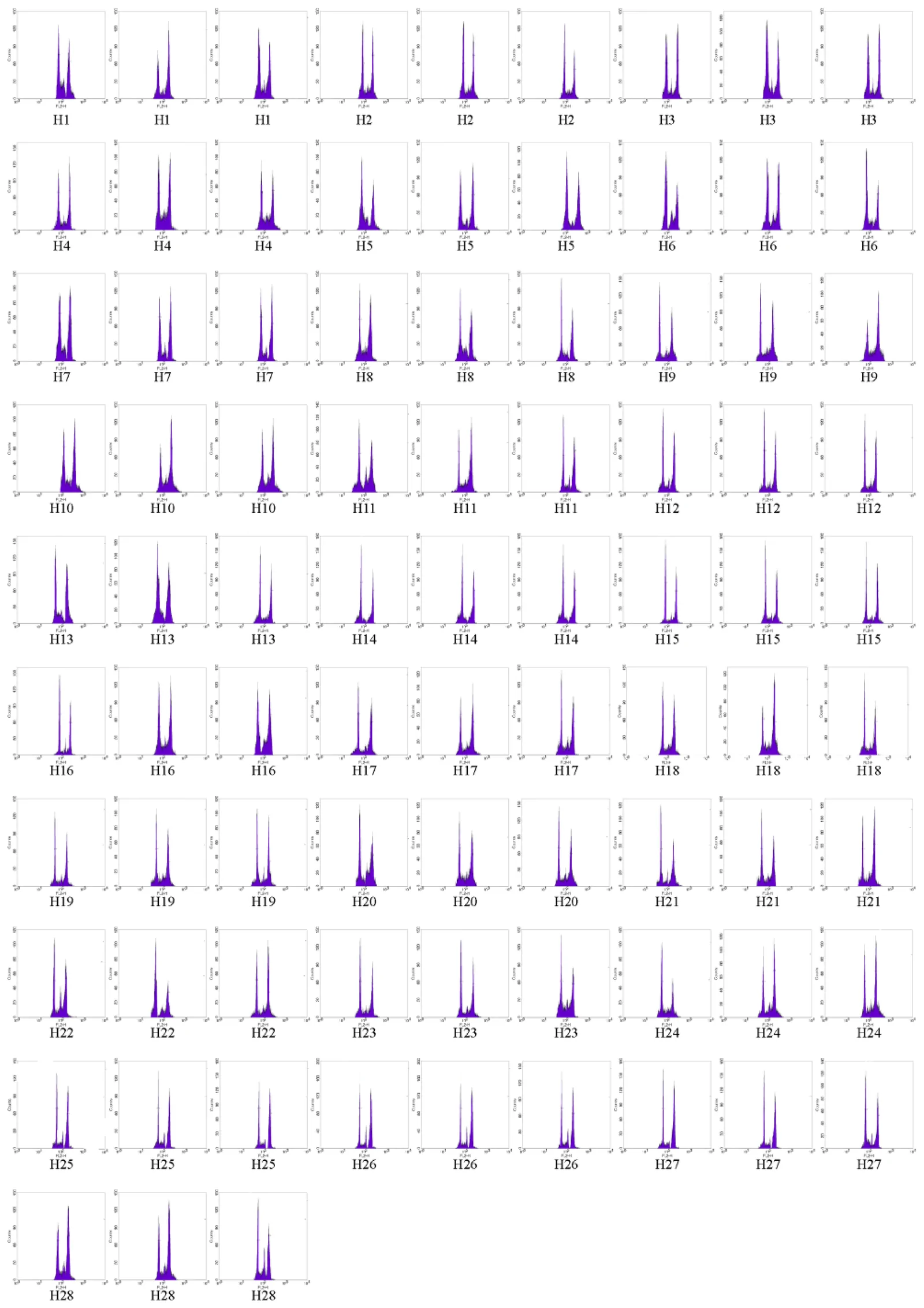
Flow cytometric histograms of 28 cultivated populations of *Paris polyphylla*.

**Figure 5:**
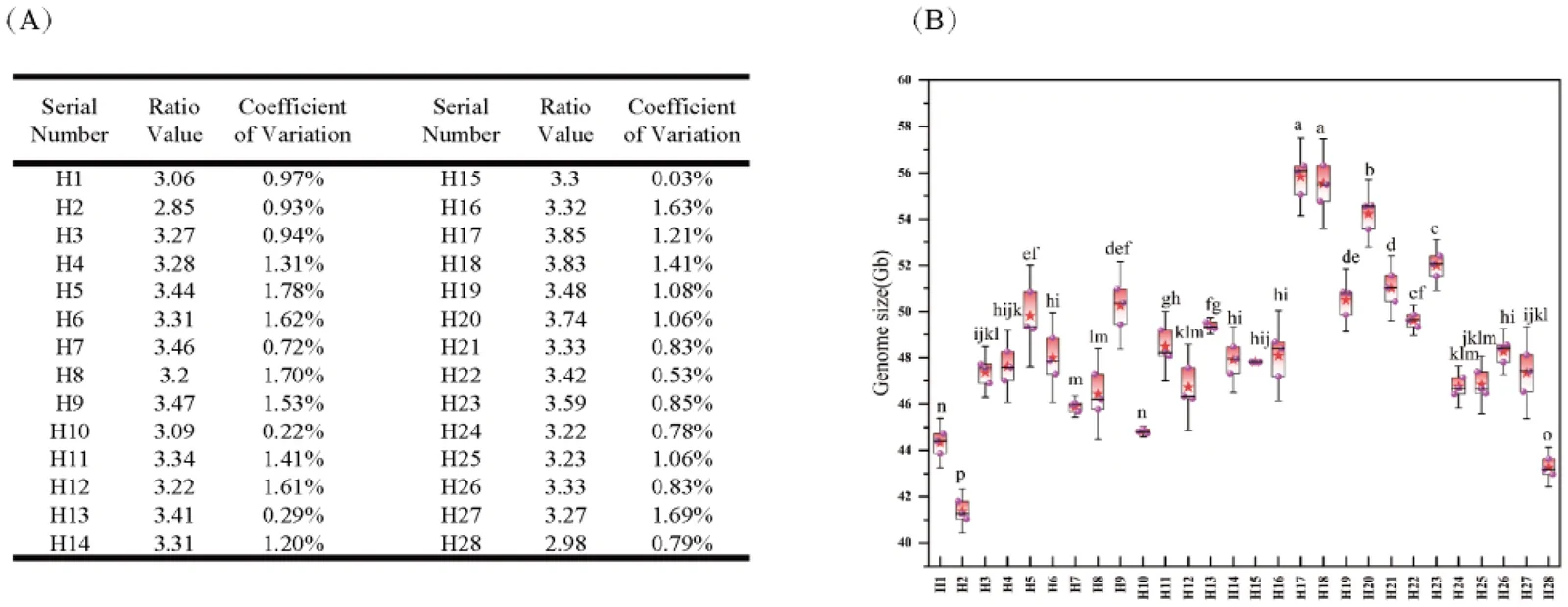
Analysis of genome size in 28 cultivated populations of *Paris* L. **(A)** Ratio and coefficient of variation of 28 cultivated populations of *Paris* L; **(B)** Box diagram of genome size of P. polyphylla var. yunnanensis in different cultivated populations. The ratio is the fluorescence intensity of the sample/the fluorescence intensity of the internal reference, and the internal reference is the China spring seed of wheat. The reference genome size is 14.5Gbp. Variation coefficient value of genome size = standard deviation of sample genome size/average value of sample genome ×100%.Data are presented as the mean ± standard deviation. Different lowercase letters indicate significant differences among populations according to one-way ANOVA followed by Tukey’s HSD test at *P*< 0.05. Significance levels are indicated as *P*< 0.05, *P* < 0.01, and *P* < 0.001.

Propidium-iodide fluorescence under 488 nm excitation yielded clear histograms for 28 populations (**Figure 4**). Using *Triticum aestivum* cv. Chinese Spring (genome size ≈ 14.5 Gbp) as internal reference, genome sizes of the 28 cultivated populations were determined (three replicates per sample). The results showed genome sizes ranged from 41.37 to 55.81 Gb (**Figure 5B**); the smallest genome size was found in H2, and the largest in H17. Significant differences in genome size were detected among populations. The coefficient of variation for genome size across populations was below 2% (**Figure 5A**), confirming measurement reliability. Flow-cytometric analysis produced clearly separated G_0_/G_1_ peaks for the samples and the internal reference standard. The estimated genome sizes of the 28 cultivated populations ranged from 41.37 to 55.81 Gb, with the smallest value detected in H2 and the largest in H17. Significant differences in genome size were observed among populations. The coefficients of variation calculated from independent genome-size measurements within each population were below 2%, indicating good technical repeatability. The mean fluorescence-intensity ratio between the sample and reference G_0_/G_1_ peaks ranged from 2.98 to 3.85 and was used to calculate relative nuclear DNA content. The diploid status of all populations was primarily confirmed by chromosome counting, which consistently showed 2n = 2x = 10.

### 3.4 Karyotype analysis among cultivated populations

**Figure 6:**
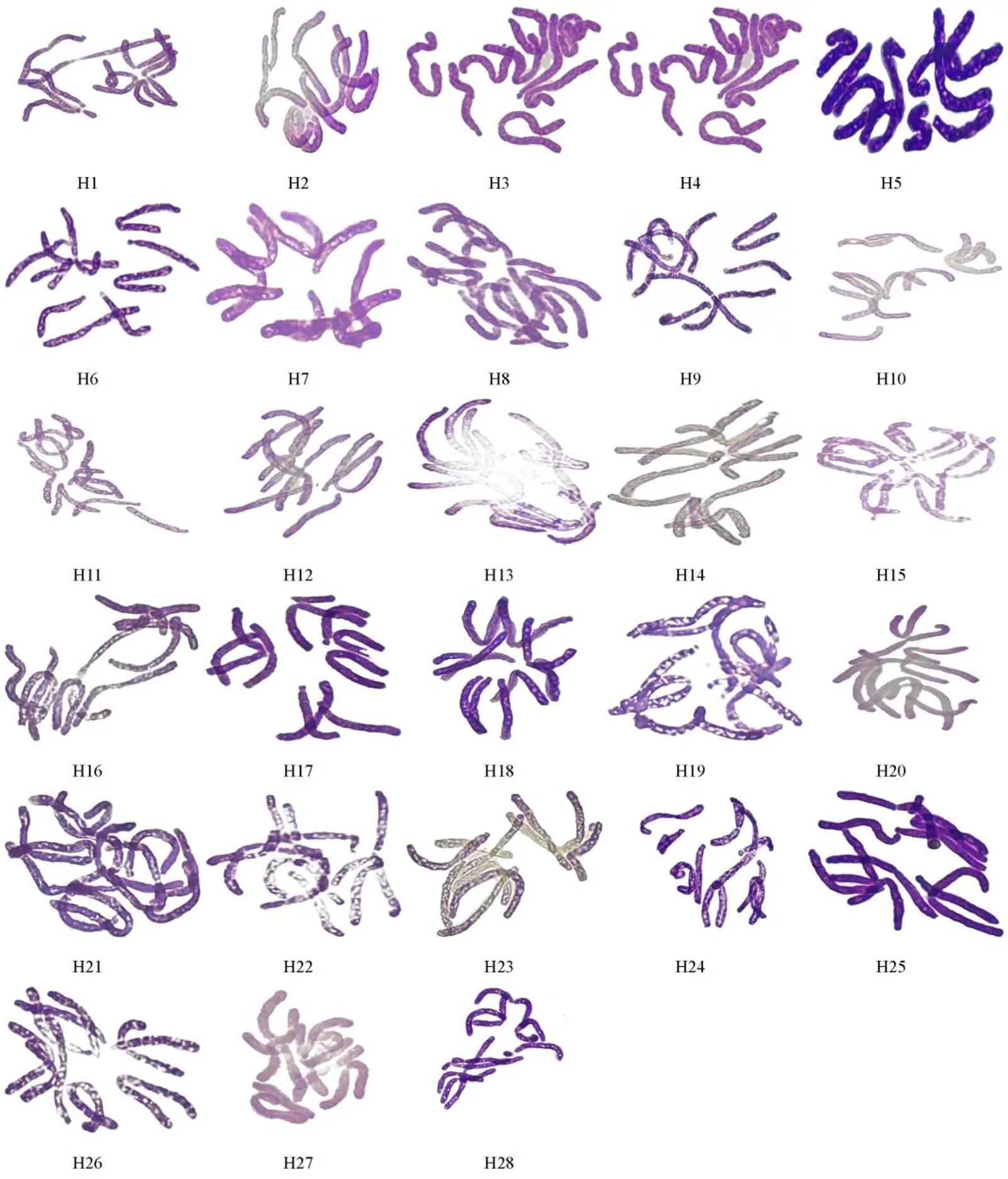
Chromosome karyotype pictures of *P. polyphylla* var. yunnanensis from different cultivated populations.

**Figure 7:**
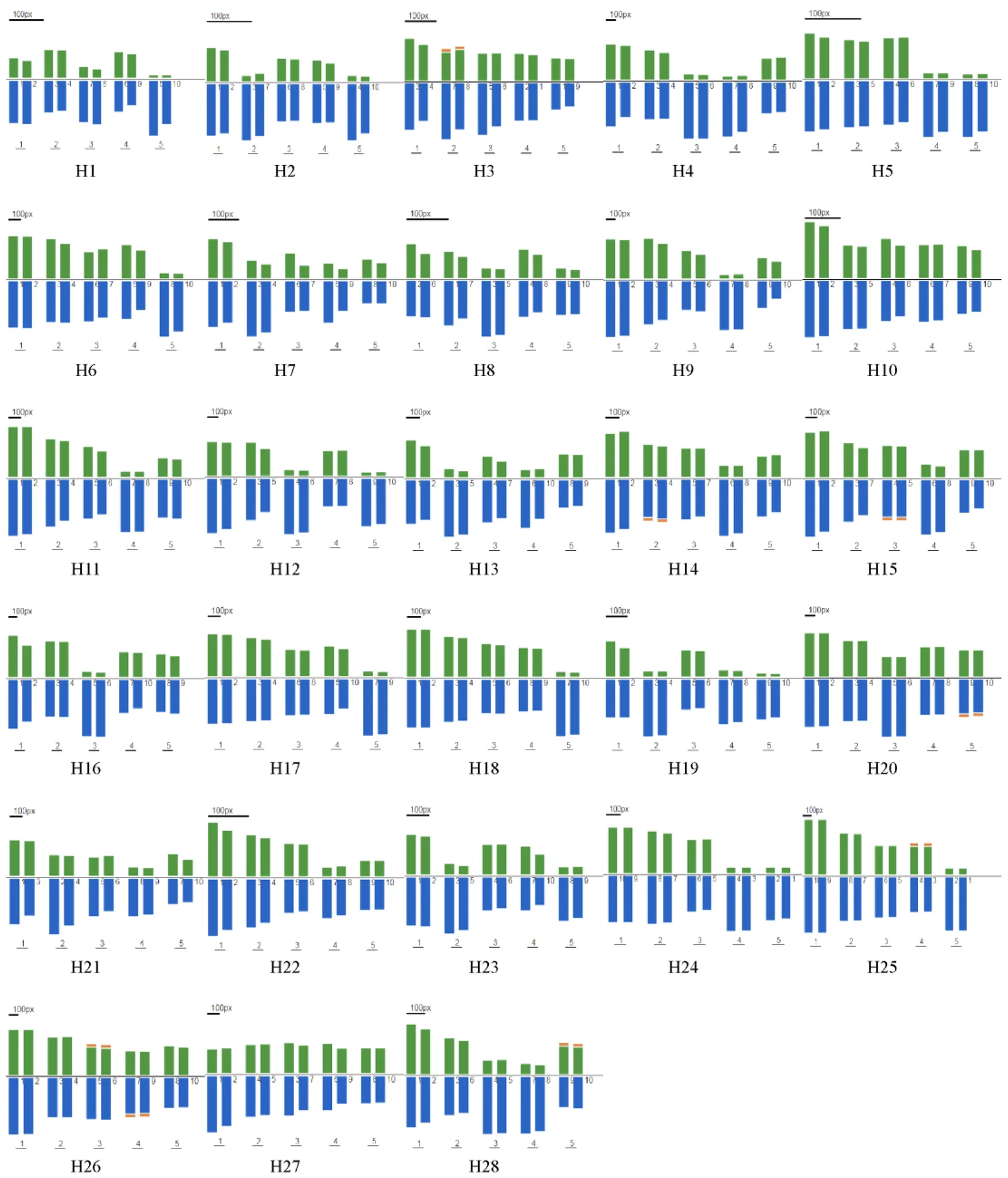
Karyotype patterns of different cultivated populations.

Chromosome spreads from metaphase cells (10 cells per sample, three individuals per population) were observed under oil-immersion (×100) and photographed (**Figure 6**). All 28 populations exhibited chromosome numbers consistent with 2n = 2x = 10 (n = x = 5), confirming diploidy. Karyotype analysis classified six asymmetry types: 1A, 2A, 3A, 1B, 2B and 3B. The predominant type was 2A (16 populations, 57%), followed by 3A (6 populations); types 1B, 2B and 3B each occurred in two populations; type 1A occurred in one (H26). Chromosome lengths ranged from 188 Mb to 614 Mb (H22 shortest, H26 longest). Centromere-index CVs ranged from 8.19% to 85.85%; chromosome-length CVs ranged from 8.1% to 36.2% (**Table 1**). Variation in chromosome-length and centromere-index coefficients of variation indicated substantial karyotypic heterogeneity among the cultivated populations. Detailed karyotype parameters (**Table 2**) show arm-ratio (long arm/short arm) varied between 1.01 and 21.56, with most values falling between 1 and 1.8, indicating predominance of median-centric chromosomes in the cultivated populations. Chromosomes were paired and measured using MATO software (**Figure 7**). Six populations (H3, H14, H20, H25, H26, H28) exhibited satellite chromosomes; H26 showed two pairs of satellites.

**Table 1:** Karyotype characteristics of *P. polyphylla* var. *yunnanensis* from 28 different cultivated populations.

Group	KF	L/S	AT	x	2n	THL	CVCI	CVCL	MCA
H1	2n = 2x = 4m + 2sm + 2st + 2t	1.36	3A	5	10	209	62.95	8.1	41.31
H2	2n = 2x = 3m + 3sm + 4t	1.56	3A	5	10	247	58.49	16.7	49.03
H3	2n = 2x = 8m(2sat) + 2sm	2.01	1B	5	10	191	13.33	21.26	16.12
H4	2n = 2x = 6m + 4t	1.54	2A	5	10	529	70.49	13.58	42.12
H5	2n = 2x = 6m + 4t	1.75	2A	5	10	323	68.73	21.34	37.72
H6	2n = 2x = 8m + 2t	1.62	2A	5	10	522	44.49	17.5	22.81
H7	2n =2x = 5m + 2sm + 3st	2.30	3B	5	10	311	30.79	30.86	30.1
H8	2n = 2x = 4m + 2sm + 4st	1.91	3A	5	10	330	42.6	17.4	36.82
H9	2n = 2x = 8m + 2t	2.78	2B	5	10	505	45.85	33.79	24.57
H10	2n = 2x = 10m	2.09	1B	5	10	366	8.19	25.06	7.79
H11	2n = 2x = 6m + 2sm + 2t	1.89	2A	5	10	552	21.69	25.96	26.56
H12	2n = 2x = 6m + 4t	1.80	2A	5	10	511	53.43	20.79	40.29
H13	2n = 2x = 4m + 2sm + 3st + 1t	1.70	3A	5	10	508	54.38	17.71	40.24
H14	2n = 2x = 7m(2sat) + 1sm + 2st	1.77	2A	5	10	383	20.96	20.84	24.29
H15	2n = 2x = 8m(1sat) + 2st(1sat)	1.83	2A	5	10	420	30.87	22.31	19.02
H16	2n = 2x = 8m + 2t	1.73	2A	5	10	480	45.58	18.31	26.02
H17	2n = 2x = 8m + 2t	1.54	2A	5	10	328	45.93	17.78	23.08
H18	2n = 2x = 8m + 2t	1.62	2A	5	10	376	46.32	21.14	19.36
H19	2n = 2x = 4m + 6t	1.81	3A	5	10	204	85.85	19.4	52.15
H20	2n = 2x = 8m(2sat) + 2sm	1.53	2A	5	10	431	22.36	16.19	16.47
H21	2n = 2x = 5m + 3sm + 2st	2.08	3B	5	10	478	34.44	26.87	32.38
H22	2n = 2x = 1M + 5m + 2sm + 2st	2.43	2B	5	10	188	31.67	36.2	20.67
H23	2n = 2x = 6m + 4st	1.83	2A	5	10	343	50.49	23.25	32.86
H24	2n = 2x = 6m + 4t	1.90	2A	5	10	490	63.79	25.35	34.07
H25	2n = 2x = 2M + 6m(2sat) + 2t	1.87	2A	5	10	422	42.85	27.37	22.47
H26	2n = 2x = 10m(4sat)	1.81	1A	5	10	614	10.59	24.78	11.3
H27	2n = 2x = 8m + 2sm	1.55	2A	5	10	462	15.38	15.42	15.31
H28	2n = 2x = 1M + 5m(2sat) + 4st	1.73	2A	5	10	312	48.53	19.69	28.55

KF, diploid karyotype formula; 2n, somatic chromosome number; x, basic chromosome number; M, chromosome with median-point centromere; m, chromosome with median-region centromere; sm, submetacentric chromosome; st, subtelocentric chromosome; t, telocentric chromosome; sat, satellite chromosome; L/S, ratio of the longest chromosome to the shortest chromosome; AT, karyotype asymmetry type; THL, total haploid chromosome length; CVCI, coefficient of variation of the centromeric index; CVCL, coefficient of variation of chromosome length; MCA, mean centromeric asymmetry.

**Table 2:** Specific karyotypes of *P. polyphylla* var. *yunnanensis* from different cultivated populations.

Group	L+S (%)	L/S	Type	Group	L+S (%)	L/S	Type
H1	15.59 + 6.62 = 22.20	2.36	sm	H15	15.03 + 12.60 = 27.62	1.19	m
	17.80 + 0.83 = 18.63	21.56	t		10.66 + 8.86 = 19.52	1.2	m
	15.49 + 3.50 = 19.00	4.42	st		10.16 + 8.67 = 18.83	1.17	st
	11.22 + 10.04 = 21.26	1.12	m		14.75 + 3.28 = 18.03	4.5	st
	10.06 + 8.85 = 18.91	1.14	m		8.43 + 7.56 = 15.99	1.12	m
H2	15.91 + 10.05 = 25.95	1.58	t	H16	13.98 + 11.27 = 25.26	1.24	m
	17.01 + 1.96 = 18.96	8.7	sm		11.37 + 10.86 = 22.23	1.05	m
	11.69 + 6.91 = 18.61	1.69	t		17.37 + 1.39 = 18.76	12.54	t
	12.28 + 6.01 = 18.29	2.04	m		9.44 + 7.52 = 16.96	1.26	m
	16.66 + 1.52 = 18.19	10.94	m		10.12 + 6.68 = 16.80	1.51	m
H3	12.19 + 11.29 = 23.48	1.08	m	H17	12.78 + 12.12 = 24.91	1.05	t
	14.81 + 8.51 = 23.33	1.74	m		11.95 + 10.74 = 22.68	1.11	t
	13.69 + 7.89 = 21.58	1.73	m		10.24 + 7.48 = 17.73	1.37	m
	10.69 + 7.52 = 18.21	1.42	m		9.25 + 8.17 = 17.42	1.13	m
	7.07 + 6.33 = 13.40	1.12	m		16.03 + 1.24 = 17.27	12.97	m
H4	12.78 + 11.22 = 24.00	1.14	m	H18	13.26 + 13.04 = 26.30	1.02	m
	11.89 + 9.03 = 20.92	1.32	m		11.48 + 10.83 = 22.32	1.06	t
	18.25 + 1.53 = 19.78	11.96	t		9.30 + 8.89 = 18.19	1.05	m
	17.47 + 1.06 = 18.52	16.53	t		8.71 + 7.92 = 16.62	1.1	m
	9.84 + 6.94 = 16.78	1.42	m		15.42 + 1.15 = 16.57	13.41	m
H5	13.23 + 11.45 = 24.69	1.16	t	H19	13.63 + 11.45 = 25.08	1.19	m
	12.21 + 9.99 = 22.20	1.22	t		20.32 + 1.84 = 22.16	11.06	t
	11.36 + 10.83 = 22.19	1.05	m		10.56 + 9.36 = 19.92	1.13	m
	14.29 + 1.37 = 15.66	10.4	m		15.74 + 2.12 = 17.85	7.43	t
	14.23 + 1.03 = 15.26	13.84	m		13.96 + 1.02 = 14.98	13.67	t
H6	13.13 + 11.88 = 25.01	1.11	m	H20	12.78 + 11.73 = 24.51	1.09	m
	11.62 + 10.40 = 22.02	1.12	m		11.30 + 9.65 = 20.95	1.17	m
	10.84 + 7.87 = 18.72	1.38	m		15.49 + 5.23 = 20.72	2.96	sm
	9.38 + 8.65 = 18.02	1.08	t		9.57 + 8.04 = 17.61	1.19	m
	14.90 + 1.33 = 16.22	11.22	t		9.08 + 7.13 = 16.21	1.27	m
H7	15.37 + 13.20 = 28.57	1.16	m	H21	14.28 + 12.05 = 26.32	1.19	sm
	18.72 + 5.48 = 24.19	3.42	m		18.06 + 6.90 = 24.96	2.62	sm
	10.62 + 6.48 = 17.10	1.64	m		12.01 + 6.59 = 18.60	1.82	m
	12.44 + 4.07 = 16.51	3.05	st		12.68 + 2.64 = 15.33	4.8	st
	7.71 + 5.91 = 13.62	1.31	m		8.43 + 6.36 = 14.79	1.33	m
H8	15.05 + 7.92 = 22.97	1.9	sm	H22	15.47 + 14.39 = 29.86	1.07	m
	12.00 + 9.70 = 21.70	1.24	m		13.22 + 11.48 = 24.69	1.15	m
	18.30 + 3.13 = 21.43	5.85	sm		9.47 + 9.29 = 18.76	1.02	M
	11.21 + 8.58 = 19.79	1.31	st		10.80 + 2.72 = 13.52	3.98	st
	11.18 + 2.93 = 14.11	3.81	st		8.68 + 4.48 = 13.16	1.94	sm
H9	17.23 + 12.15 = 29.38	1.42	m	H23	15.32 + 12.31 = 27.63	1.24	m
	12.77 + 11.63 = 24.39	1.1	m		17.20 + 3.06 = 20.25	5.63	m
	9.21 + 8.07 = 17.28	1.14	m		9.95 + 9.46 = 19.41	1.05	m
	15.16 + 1.16 = 16.32	13.12	t		9.54 + 7.60 = 17.14	1.25	st
	6.85 + 5.78 = 12.63	1.19	m		13.22 + 2.35 = 15.57	5.62	m
H10	14.61 + 13.86 = 28.47	1.05	m	H24	12.89 + 12.63 = 25.52	1.02	t
	11.94 + 7.91 = 19.85	1.51	m		13.24 + 11.46 = 24.70	1.16	t
	9.37 + 8.84 = 18.20	1.06	m		9.79 + 9.39 = 19.18	1.04	m
	9.96 + 8.21 = 18.17	1.21	m		15.43 + 1.51 = 16.94	10.25	m
	7.92 + 7.38 = 15.30	1.07	m		12.15 + 1.51 = 13.66	8.03	m
H11	15.15 + 13.74 = 28.90	1.1	m	H25	12.89 + 12.63 = 25.52	1.02	t
	13.54 + 5.89 = 19.43	2.3	m		13.24 + 11.46 = 24.70	1.16	t
	12.83 + 5.72 = 18.55	2.24	m		9.79 + 9.39 = 19.18	1.04	m
	10.49 + 4.94 = 15.43	2.12	t		15.43 + 1.51 = 16.94	10.25	m
	10.11 + 7.59 = 17.70	1.33	sm		12.15 + 1.51 = 13.66	8.03	m
H12	17.43 + 6.20 = 23.62	2.81	m	H26	15.51 + 12.44 = 27.96	1.25	m
	14.55 + 10.52 = 25.07	1.38	t		10.78 + 10.44 = 21.23	1.03	m
	13.80 + 5.07 = 18.88	2.72	t		11.42 + 7.40 = 18.82	1.54	m
	14.73 + 1.05 = 15.79	13.98	m		9.70 + 6.45 = 16.15	1.5	m
	8.73 + 7.91 = 16.64	1.1	t		8.11 + 7.73 = 15.84	1.05	m
H13	13.99 + 11.47 = 25.46	1.22	m	H27	16.55 + 7.60 = 24.15	2.18	sm
	18.64 + 2.39 = 21.03	7.81	sm		12.38 + 8.91 = 21.29	1.39	m
	13.47 + 6.21 = 19.67	2.17	st		11.45 + 9.02 = 20.46	1.27	m
	14.60 + 2.63 = 17.23	5.55	m		9.60 + 8.33 = 17.93	1.15	m
	8.94 + 7.67 = 16.61	1.17	st		8.42 + 7.75 = 16.17	1.09	m
H14	9.80 + 5.98 = 15.78	1.64	m	H28	13.36 + 13.18 = 26.54	1.01	m
	10.80 + 8.87 = 19.68	1.22	m		10.21 + 9.57 = 19.78	1.07	m
	14.44 + 12.51 = 26.96	1.15	st		15.76 + 3.82 = 19.57	4.13	st
	13.54 + 5.63 = 19.17	2.4	st		15.40 + 2.56 = 17.96	6.02	st
	12.80 + 5.61 = 18.41	2.28	m		8.59 + 7.56 = 16.15	1.14	m

M, Centromere chromosome, m, Centromere chromosome, Sm, Proximal Centromere chromosome, St, Proximal Centromere chromosome, t, Terminal Centromere chromosome.

### 3.5 Saponin content among cultivated populations

**Figure 8:**
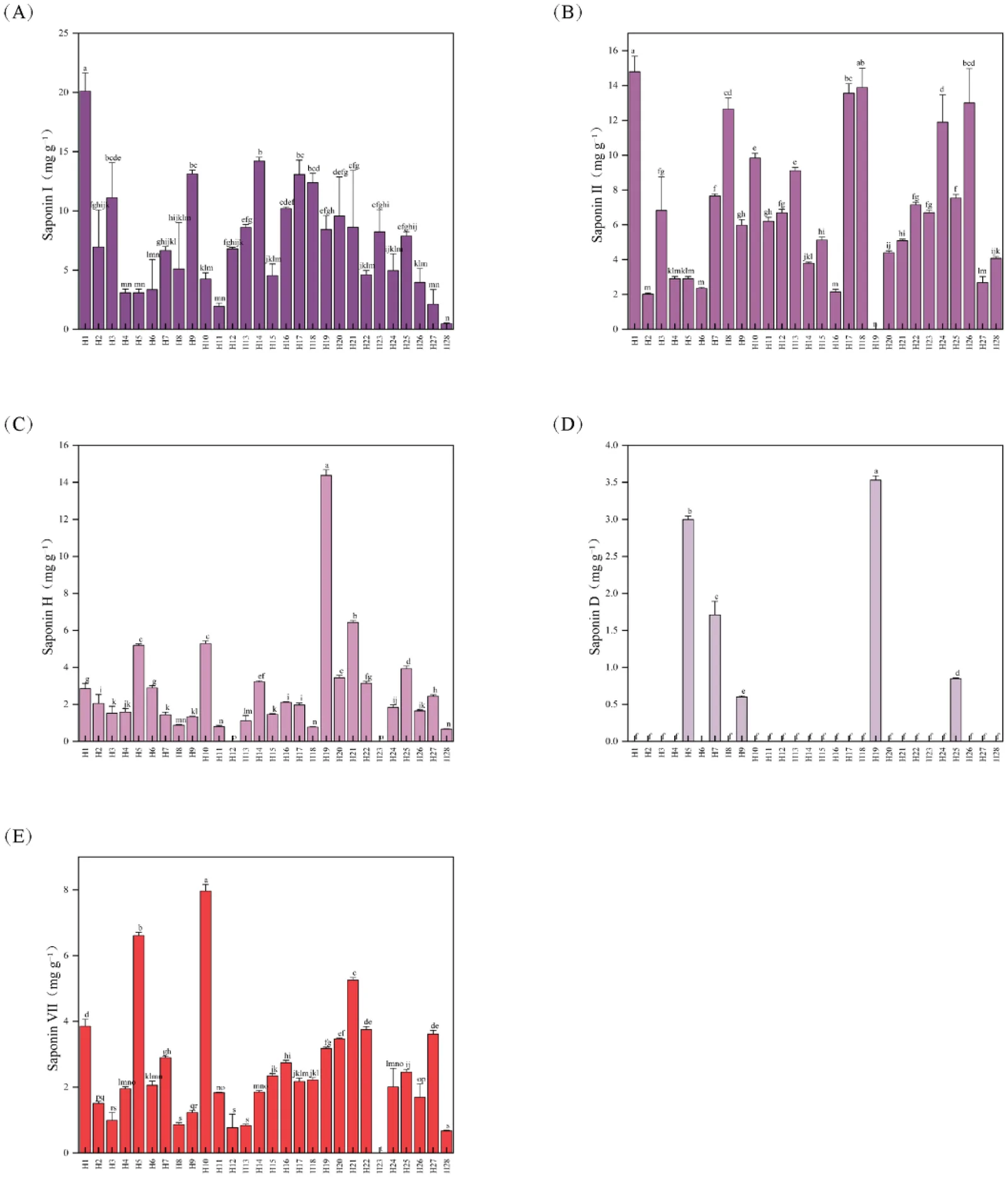
Saponin content chart of 28 cultivated populations of *Paris polyphylla*. **(A)** Saponin I; **(B)** Saponin II; **(C)** Saponin H; **(D)** Saponin D; **(E)** Saponin VII.

To investigate phytochemical variation, HPLC quantified six steroidal saponins (polyphyllin I, II, VI, VII, D, H) in Rhizomes from the 28 populations (**Figure 8**). Polyphyllin VI was not detected in any cultivated-population sample under the current HPLC conditions. In contrast, the mixed reference-standard chromatograms showed a well-resolved polyphyllin VI peak at approximately 21.9–22.0 min, demonstrating that the chromatographic method was capable of detecting and separating this compound. Therefore, the absence of a detectable peak in the analyzed samples should not be interpreted as definitive evidence that polyphyllin VI was completely absent. Instead, the compound may have been present at concentrations below the analytical detection capability of the method. Saponin D was detected only in five populations (H5, H7, H9, H19, H25) (**Figure 8D**). All populations contained saponin I (**Figure 8A**). Population H19 lacked saponin II (**Figure 8B**); H12 and H13 lacked saponin H (**Figure 8C**); H23 lacked saponin VII (**Figure 8E**). The highest contents for saponin I and II were in H1 (20.11 mg g^-1^) and H1 (14.79 mg g^-1^), respectively; the lowest for saponin I and II were in H28 (0.49 mg g^-1^) and H19 (0 mg g^-1^), respectively. Saponin H was highest in H19 (14.38 mg g^-1^) and zero in H12 and H23; saponin D peaked at 3.53 mg g^-1^ in H19; saponin VII peaked at 7.96 mg g^-1^ in H10 and was zero in H23. These data demonstrate significant differences in saponin profiles among cultivated populations of *P. polyphylla* in Yunnan.

### 3.6 Correlation analysis among saponin content, agronomic traits, karyotype parameters, and genome size

**Figure 9:**
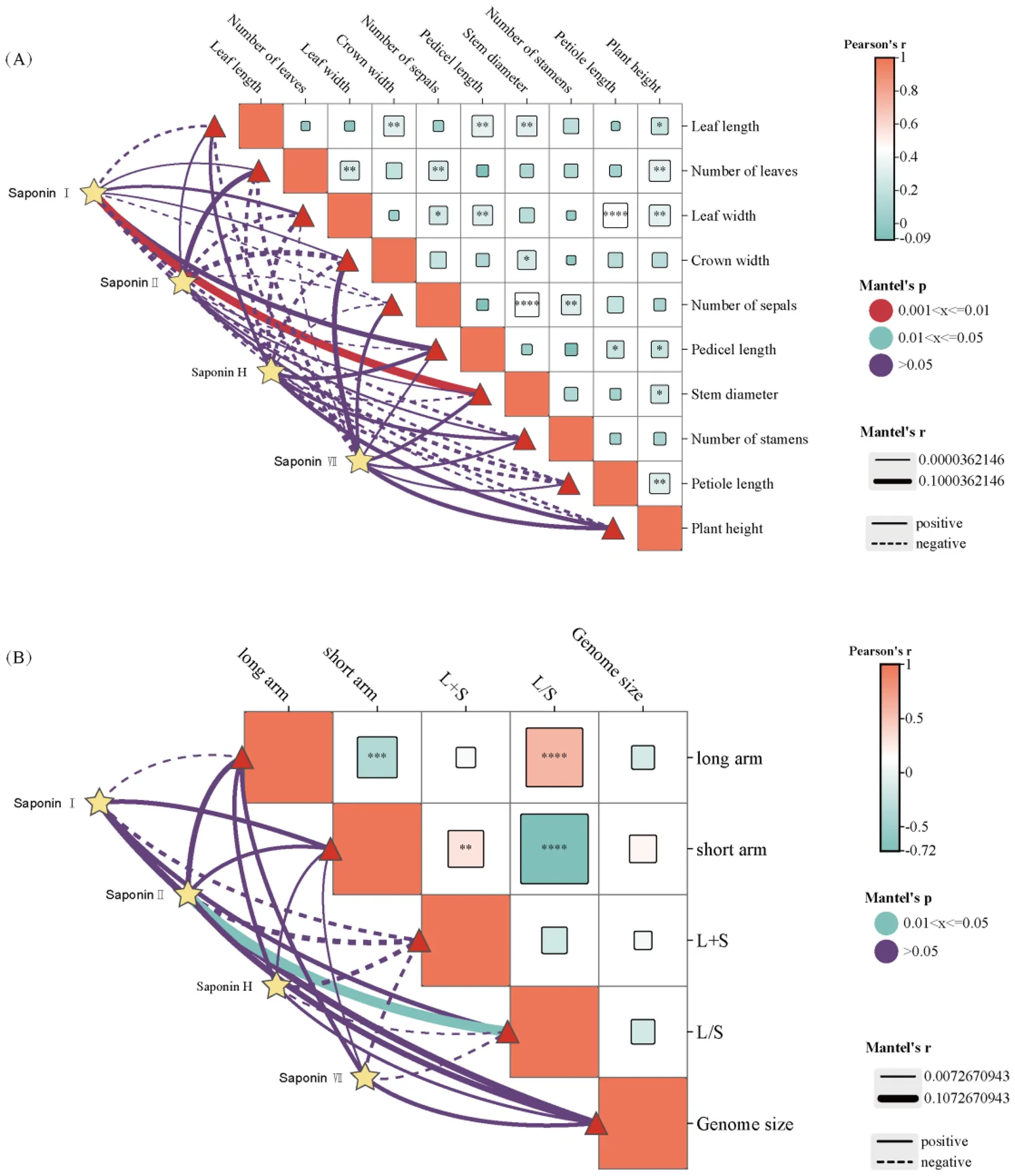
Correlation between saponins and **(A)** agronomic traits, **(B)** chromosome karyotype, and genome size. Data are presented as the mean ± standard deviation. Different lowercase letters indicate significant differences among populations according to one-way ANOVA followed by Tukey’s HSD test at *P* < 0.05. Significance levels are indicated as *P < 0.05, **P < 0.01, ***P < 0.001, and ****P < 0.0001.

Pearson correlation analysis together with Mantel tests was conducted (**Figure 9**). Saponin I was significantly positively correlated with stem diameter and positively correlated with leaf number, leaf width, and crown width. Saponin II correlated positively with leaf length, leaf number, and stamen number. Saponin H correlated positively with leaf length, peduncle length and plant height. Saponin VII correlated positively with crown width, sepal number, peduncle length, stem diameter, stamen number, petiole length and plant height (**Figure 9A**). Regarding chromosomal and genomic parameters, saponin I correlated positively with short-arm length, long/short arm ratio and genome size. Saponin II correlated significantly with long/short arm ratio and positively with long arm length, short arm length, and genome size. Saponin H correlated positively with long arm, short arm and genome size. Saponin VII correlated positively with long arm, short arm and genome size (**Figure 9B**).

### 3.7 Factors influencing growth and quality of different cultivated populations

**Figure 10:**
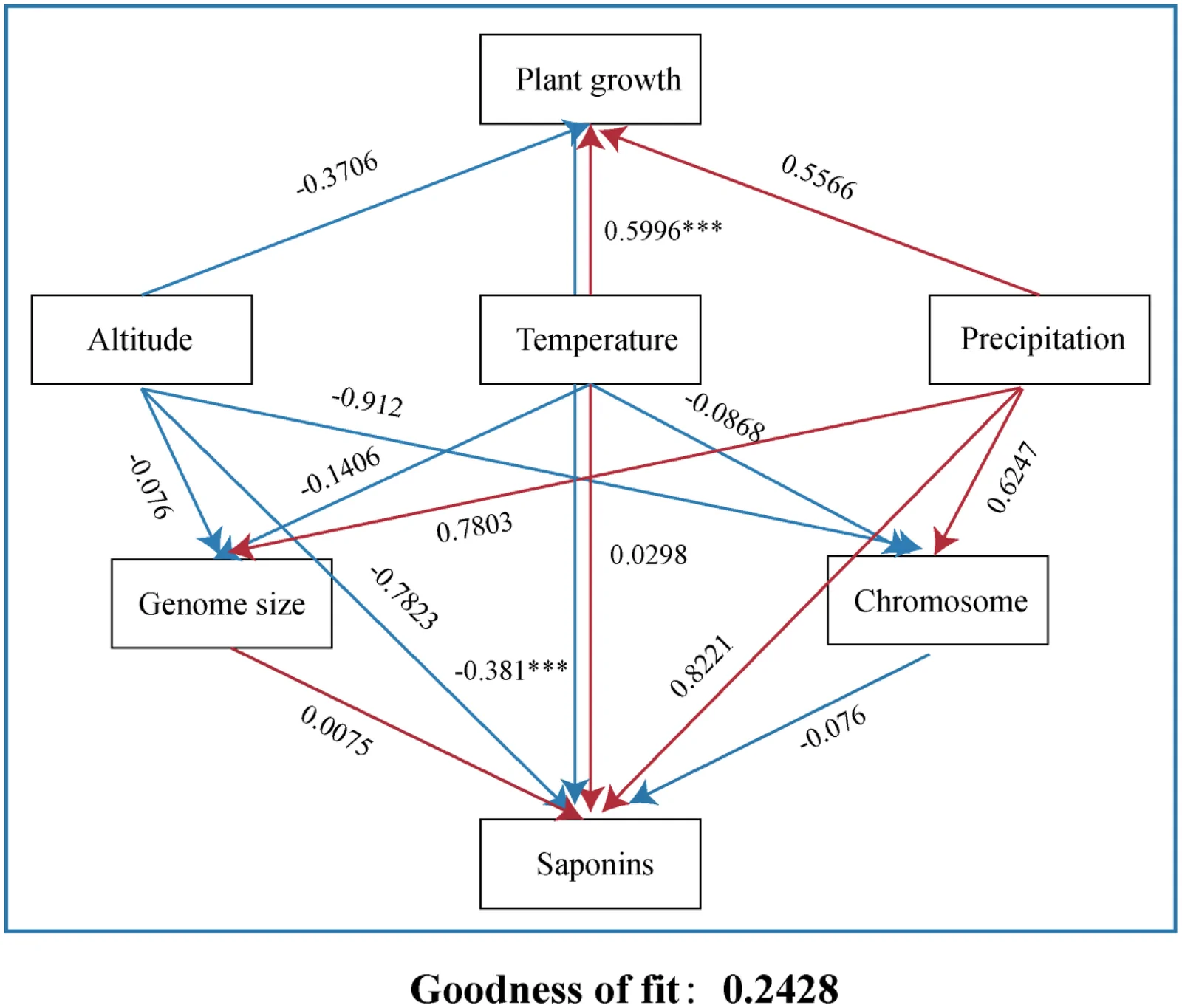
Exploratory partial least squares path model showing associations among source-site altitude, mean annual temperature, mean annual precipitation, karyotypic characteristics, genome size, plant-growth traits, and saponin profiles. Values adjacent to arrows represent standardized path coefficients. The R² values for the karyotype construct, genome size, plant-growth construct, and saponin-quality construct were 0.080, 0.096, 0.222, and 0.168, respectively, and the global goodness-of-fit value was 0.2428. Red and blue arrows indicate positive and negative path coefficients, respectively. The significance values were derived from the internal linear-model estimates rather than bootstrap resampling; therefore, the paths should be interpreted as exploratory statistical associations. ***P < 0.001.

The PLS-PM had a global goodness-of-fit value of 0.2428 (**Figure 10**). The coefficients of determination for the karyotype construct, genome size, plant-growth construct, and saponin-quality construct were 0.080, 0.096, 0.222, and 0.168, respectively. Thus, the environmental variables explained 8.0% of the variation in the karyotype construct, 9.6% of the variation in genome size, and 22.2% of the variation in the plant-growth construct. The complete model explained 16.8% of the variation in the saponin-quality construct, indicating low-to-moderate explanatory capacity.

Among the estimated paths, temperature was positively associated with the plant-growth construct (path coefficient = 0.600, P = 0.0023), whereas the plant-growth construct was negatively associated with the saponin-quality construct (path coefficient = −0.381, P = 0.0018). The direct association between temperature and the saponin-quality construct was weak and not statistically significant (path coefficient = 0.0298, P = 0.889).

The remaining direct paths did not reach the nominal significance threshold of P< 0.05. Because the model was based on repeated measurements nested within 28 populations and was not validated using bootstrap resampling or a reliable cross-validation index, these results should be interpreted as exploratory associations rather than causal effects.

## 4 Discussion

In this study, we compared the phenological and morphological variations among 28 cultivated populations of *Paris polyphylla* var. *yunnanensis* and found that key developmental stages—including sprouting, flowering, fruit dehiscence, and senescence—were largely synchronized but exhibited distinct temporal shifts among populations, particularly in H2, where senescence was markedly delayed until the following February (**Figure 1B**). Such deviations indicate divergent developmental rhythms and adaptive strategies among cultivated populations, likely shaped by variations in temperature, photoperiod, and altitude (Thakur et al., 2023; Wang et al., 2024; Yan et al., 2024). For perennial medicinal plants, autumn warming can delay leaf senescence and extend the growing season, thereby altering carbon allocation and metabolic processes (Chen and Zhang, 2023). This provides a plausible ecophysiological explanation for the delayed senescence and extended growth period observed in H2. Moreover, interactions between temperature and light intensity have been shown to regulate phenological progression and secondary metabolism in *Panax* species (Chen et al., 2022), suggesting that differential sensitivity to local microclimates may underlie the observed phase displacements among populations.

Morphologically, all populations shared a lanceolate leaf shape, indicating a high degree of conservatism within the species. However, the coefficients of variation (CVs) of ten quantitative traits revealed substantial differences in stability (**Figure 2**): petiole length and stamen number exhibited the highest variability, while leaf length in population H11 showed exceptional uniformity (CV = 0.74%), reflecting strong genetic homogeneity. Quantitative traits are generally more plastic and environmentally sensitive, a pattern frequently reported for alpine medicinal plants responding to gradients in elevation, temperature, and moisture (Sharma et al., 2024; Yang et al., 2025; Zhou et al., 2024). In breeding and population evaluation, materials with low trait CVs (e.g., H11) are often regarded as ideal sources for stable cultivation and standardization (Abaya et al., 2024; Ma et al., 2024), whereas highly variable traits (e.g., petiole length) can serve as sensitive indicators for distinguishing populations and detecting environmental stress in multi-location trials.

DNA barcoding, as a robust tool for species identification, has demonstrated tremendous potential in medicinal plant authentication. Ideal barcode regions should possess sufficient variability for discrimination while maintaining conserved flanking regions for primer design (Hebert and Gregory, 2005). In this study, the nuclear ITS and chloroplast trnL–trnF regions both exhibited high amplification (100%) and sequencing success (>98.5%), meeting the key criteria for effective barcoding (Chen et al., 2010). In the present study, ITS exhibited greater haplotype richness and sequence variation among the representative cultivated-population sequences than *trnL–trnF*. However, because each population was represented by only one consensus sequence, this observation should be interpreted as a comparison of marker resolution within the present dataset rather than a formal assessment of population-level discriminatory success. The absence of replicate sequences within populations prevented estimation of within-population genetic distances and conventional DNA barcode gap analysis. Future studies should include multiple individuals per population to quantitatively compare within- and among-population variation and to evaluate marker performance more rigorously. These differences are consistent with the distinct evolutionary characteristics of nuclear ribosomal ITS and chloroplast non-coding regions reported in previous studies (Álvarez and Wendel, 2003; Shaw et al., 2007). The ITS region showed a higher G+C content and greater haplotype richness, whereas trnL–trnF showed a higher A+T content and fewer haplotypes. However, these differences should be interpreted as differences in sequence-variation patterns and marker performance in the present dataset rather than as evidence that trnL–trnF is inherently more conserved or more stable than ITS. Together, the two barcode regions provided complementary information for distinguishing the cultivated populations examined in this study. Fifteen ITS haplotypes (Hd = 0.884) were detected, compared with only five *trnL–trnF* haplotypes (Hd = 0.603) (**Figure 3D**), likely reflecting the complex evolutionary history of the genus, where nuclear genomes record extensive hybridization and gene flow, while chloroplast genomes retain conservative maternal signals (Li et al., 2021a). The high genetic diversity observed suggests that cultivated *P. polyphylla* populations still harbor rich genetic resources, which is crucial for germplasm conservation (Deng et al., 2015). The well-supported ITS clades trnL–trnF (e.g., H2–H25, 100% bootstrap) (**Figure 3A**) indicate multiple genetic lineages within current cultivars (Liang et al., 2019). In contrast, several internal branches of the *trnL–trnF* NJ tree received low bootstrap support, indicating that relationships among some representative population sequences remained unresolved. Collectively, these findings provide molecular evidence for the domestication and cultivation history of *P. polyphylla*. Under the present sampling design, ITS provided greater sequence resolution among the representative cultivated-population sequences, whereas *trnL–trnF* provided complementary chloroplast information. Nevertheless, broader sampling involving multiple individuals per population and additional related taxa is required before either marker can be recommended for formal population- or species-level identification. *Paris polyphylla*, an important medicinal resource, is currently threatened by overharvesting and habitat degradation. The high genetic diversity revealed in this study underscores the urgency of conserving its wild populations. DNA barcoding serves not only as an effective tool for authenticating medicinal materials but also for tracing the geographical origins of wild populations, thereby providing a scientific basis for formulating conservation strategies (Gong et al., 2016). Moreover, genetic diversity data can guide the breeding of superior cultivars, promoting the sustainable utilization of this valuable resource. Although haplotypes were identified from the representative sequences, a population-level haplotype network was not constructed because only one consensus sequence was available for each population. Consequently, the present dataset could not characterize within-population haplotype frequencies or adequately represent the distribution of haplotypes among individuals. Future studies incorporating multiple individuals per population will be required to construct a robust population-level haplotype network.

Flow cytometry is a powerful cytogenetic approach that enables accurate estimation of genome size and ploidy level in plants (Greilhuber et al., 2005). Previous studies using flow cytometry in the tribe *Lilieae* demonstrated variations in haploid genome size among 23 species, and such changes have been linked to lineage diversification. This supports the hypothesis that a high diversification rate may benefit from genome size variation through mechanisms such as polyploidy and genomic responsiveness (Puttick et al., 2015; Sassone et al., 2018). Similarly, flow cytometric analyses of the genus *Sorbus* revealed that genome size variation among eight native Chinese species could be used to infer their ploidy levels (Li et al., 2021b).

In the present study, all *P. polyphylla* samples exhibited distinct G_0_/G_1_ peaks with coefficients of variation (CVs) below 2% (**Figure 5A**), indicating high data quality and technical reproducibility. Low CV values are widely recognized as a critical indicator of reliable flow cytometric measurements (Nix et al., 2024; Sliwinska et al., 2022). The estimated genome sizes of the 28 cultivated populations ranged from 41.37 GB to 55.81 GB (**Figure 5B**), showing significant inter-population differences. The relative fluorescence ratios (2.98–3.85) consistently supported their diploid status. These findings are consistent with previous reports that *Paris* species possess extraordinarily large genomes, a phenomenon attributed to the massive accumulation of repetitive elements such as LTR retrotransposons (Hua et al., 2022).

Notably, although all populations possessed the same chromosome number and diploid level, their estimated nuclear genome sizes differed substantially. This pattern is not necessarily contradictory because ploidy and genome size describe different genomic properties. Ploidy refers to the number of complete chromosome sets, whereas genome size refers to the total amount of nuclear DNA. Considerable variation in the abundance of DNA sequences within chromosomes can therefore occur without altering chromosome number. The range of 41.37–55.81 Gb observed in the present study may consequently reflect differences in chromosome-level DNA composition rather than changes in whole-genome ploidy.

The differential proliferation and removal of repetitive DNA may provide a plausible molecular explanation for this variation. Repetitive sequences are major determinants of plant genome size, and transposable elements can account for a substantial proportion of large plant genomes. Among them, long-terminal-repeat retrotransposons can increase their copy number through an RNA-mediated copy-and-paste mechanism, leading to genome expansion without necessarily changing chromosome number (He et al., 2024; Lee and Kim, 2014). Conversely, unequal homologous recombination, illegitimate recombination, small deletions, and double-strand-break repair can remove repetitive sequences and contribute to genome contraction (He et al., 2024). In addition to transposable elements, variation in satellite DNA, tandem repeats, ribosomal-DNA arrays, and other heterochromatin-associated repetitive sequences may contribute to differences in nuclear DNA content among populations. Importantly, genomic analyses of *Paris polyphylla* var. *yunnanensis* have shown that its exceptionally large genome contains a high proportion of repetitive sequences and LTR retrotransposons, supporting the hypothesis that repeat accumulation has played a central role in genome expansion in this lineage (Zeng et al., 2025).

Nevertheless, the present study did not include whole-genome sequencing, repeat annotation, or fluorescence *in situ* hybridization for the 28 populations. Therefore, differential transposable-element proliferation and repetitive-DNA expansion should be regarded as plausible mechanisms rather than directly demonstrated causes of the observed genome-size variation. Future long-read genome sequencing, low-coverage repeatome profiling, estimation of LTR insertion times, and repeat-based fluorescence *in situ* hybridization would be valuable for identifying the specific sequence components responsible for genome expansion or contraction among populations.

Potential technical variation associated with flow cytometry should also be considered. Flow-cytometric estimates of plant nuclear DNA content can be was associated with by leaf developmental stage, tissue type, nuclei-isolation efficiency, extraction-buffer composition, staining conditions, secondary metabolites, the choice of reference standard, and instrument settings (Doležel et al., 2007; Nix et al., 2024; Temsch et al., 2021). Paris polyphylla contains abundant steroidal saponins, phenolic compounds, polysaccharides, and other secondary metabolites that may interfere with nuclei release, increase background debris, or affect stoichiometric propidium-iodide staining. Moreover, an internal standard with a genome size markedly different from that of the target sample may increase the risk of nonlinearity or offset errors, and the use of different analytical batches may introduce additional instrument-related variation (Nix et al., 2024).

In the present study, young leaves of comparable developmental stages were processed using standardized extraction and staining procedures, and the coefficients of variation were maintained within an acceptable range. These results support good technical repeatability. However, low CV values primarily indicate precision and peak quality and do not completely exclude systematic errors associated with the reference standard, sample-specific matrix effects, or instrument calibration. Thus, the observed range of 41.37–55.81 Gb may comprise both genuine biological variation and a component of technical variation. Future studies should use co-processing and co-staining of the sample and reference standard, repeat measurements across analytical dates, and, where possible, more than one reference standard. Independent estimates based on genome-survey sequencing or k-mer analysis would also help validate the flow-cytometric results.

Genome-size variation may have important biological and ecological consequences. Through nucleotypic effects, genome size can influence nuclear and cell volume, cell-division rate, stomatal size and density, tissue-development rate, and the duration of DNA replication. Larger plant genomes are frequently associated with larger cells, slower cell division, reduced maximum growth rates, and greater nitrogen and phosphorus investment in nucleic acids, potentially influencing plant growth strategies and responses to resource limitation (Guignard et al., 2016; Roddy et al., 2020). Repetitive sequences and transposable elements may also affect chromatin organization, gene regulation, recombination, and stress-responsive genomic plasticity. Genome-size variation among the cultivated populations may therefore reflect genomic differentiation associated with source-environment adaptation, cultivation history, or artificial selection.

Positive associations were detected between genome size and several saponin components in the present study. However, these correlations should not be interpreted as evidence that genome expansion directly promotes saponin biosynthesis. Most DNA added during the expansion of large plant genomes consists of non-coding repetitive sequences, and a larger genome does not necessarily contain more protein-coding genes or more copies of saponin-biosynthesis genes. The observed associations may instead reflect shared effects of population genetic background, chromosome structure, plant developmental status, and source-site environmental conditions. Genome size may therefore serve as a complementary indicator of germplasm differentiation, but its usefulness for predicting medicinal quality requires further validation through repeat-content analysis, gene-copy-number assessment, and multi-environment experiments.

Chromosome analysis showed that all 28 cultivated populations possessed the same chromosome number (2n = 2x = 10) but differed in chromosome morphology, centromere position, karyotype category, and the occurrence of satellite chromosomes, indicating cytogenetic heterogeneity despite the conserved chromosome number.

Karyotype characteristics, including chromosome size, morphology, and centromere position, provide useful descriptive information for comparing cytogenetic variation among taxa. However, these characteristics should not be interpreted as direct indicators of evolutionary status. Contemporary comparative cytogenetic studies emphasize that the evolutionary direction of chromosome changes should be evaluated by mapping cytogenetic characters onto a well-resolved molecular phylogeny and, where possible, reconstructing their ancestral states (Deanna et al., 2022; Ibiapino et al., 2022; Wolny et al., 2025). Although the classical Stebbins framework distinguished relatively symmetrical and asymmetrical karyotypes (Stebbins, 1971), such categories alone cannot establish ancestral–derived relationships or evolutionary rank.

Therefore, the six karyotype categories identified in the present study should be interpreted as evidence of chromosome morphological variation rather than differences in evolutionary status. The observed variation may be associated with chromosome rearrangements, changes in repetitive-DNA abundance, or differences in heterochromatin distribution. However, because these genomic features were not directly examined, they remain plausible explanations rather than demonstrated mechanisms. Modern molecular cytogenetic approaches, including chromosome-specific and repeat-based fluorescence *in situ* hybridization, would be required to characterize these structural differences more accurately (Wolny et al., 2025).

Variation in satellite chromosomes may similarly reflect differences in nucleolar organizer regions and ribosomal-DNA distribution. However, the number and morphology of satellites visible after conventional chromosome staining do not necessarily correspond directly to the actual number, activity, or chromosomal distribution of rDNA loci. Consequently, fluorescence *in situ* hybridization using 5S and 35S/45S rDNA probes would be required to characterize these regions and avoid overinterpreting conventional satellite morphology (Mitrenina et al., 2023; Rosselló et al., 2022).

Because the ITS and trnL–trnF markers used in this study were intended primarily for germplasm identification rather than high-resolution phylogenetic reconstruction, the present data do not provide sufficient evidence to infer the direction of karyotype evolution. Instead, the observed differences are more appropriately interpreted as cytogenetic differentiation among cultivated populations.

Steroidal saponins are major pharmacologically active constituents of *P. polyphylla*, known for their anticancer, anti-inflammatory, and cardioprotective properties (Challinor and De Voss, 2013; Guo, 2016; Newman and Cragg, 2016; Podolak et al., 2010). HPLC quantification of six key saponins (I, II, VI, VII, D, H) revealed marked compositional divergence among populations (**Figure 8**). All populations contained saponin I, while saponin VI was absent in all samples; saponin D occurred only in five populations (H5, H7, H9, H19, H25). Some populations exhibited the loss or enrichment of specific saponins (e.g., absence of II in H19, absence of H in H12 and H13, absence of VII in H23), reflecting population-specific chemotypes rather than random variation. Similar multicomponent and heterogeneous chemical diversity has been reported in other *Paris* species and represents a key biological feature of the genus (Sun et al., 2025; Xu et al., 2024). The differentiation in saponin profiles likely results from multilayered factors, including genotypic structure, allelic variation, and environmental regulation of biosynthetic pathways. The co-occurrence of high D and H but absence of II in H19 suggests a possible genotype–metabolite linkage. Environmental and microbial interactions have also been implicated, as soil nutrients and rhizosphere microbiota composition were shown to correlate positively with total saponin and specific monomers (I, II) (Liu et al., 2024), while shaded forest habitats promote accumulation of steroidal saponins and polyphenols (Yan et al., 2024). The contents of the major steroidal saponins detected in the present study, ranging from undetectable levels to 20.11 mg g^-1^, were comparable in magnitude to those reported in previous studies of *Paris polyphylla*. For example, substantial differences in polyphyllin I, II, and VII contents have also been reported among *P. polyphylla* samples from different geographical origins, indicating that pronounced phytochemical variation is a common feature within this species (Shu et al., 2025; Xu et al., 2024). These findings emphasize the coordinated regulation of secondary metabolism by both genetic and ecological factors. The present HPLC analysis detected five major steroidal saponins in the cultivated populations, whereas polyphyllin VI was not detected under the applied analytical conditions. Because the mixed reference standards produced a well-resolved polyphyllin VI peak, its non-detection was unlikely to result from chromatographic failure, although trace amounts below the analytical detection capability cannot be excluded. A limitation of this study is that complete analytical-validation records, including calibration curves, linear ranges, LOD/LOQ values, and spike-recovery data, were unavailable because the quantitative analysis had been completed previously using a routine analytical service. Therefore, the absence of detectable polyphyllin VI should be interpreted cautiously.

Pearson correlation analysis identified statistical associations between several saponin components and selected agronomic, karyotypic, and genome-size variables (**Figure 9**). In particular, genome size was positively correlated with the contents of several saponins. However, these correlations do not demonstrate that genome expansion directly promotes steroidal-saponin biosynthesis. Variation in plant genome size is frequently driven by the expansion or removal of transposable elements and other non-coding repetitive sequences and does not necessarily correspond to proportional increases in protein-coding gene number or in the copy number of genes involved in secondary metabolism (He et al., 2024; Yuan et al., 2024).

The observed associations may instead reflect shared variation in population genetic background, chromosome structure, plant developmental status, cultivation history, or environmental conditions at the source sites. Unmeasured variables may also have contributed to the correlations. In observational studies, statistical associations may be affected by measured and unmeasured confounding factors and therefore cannot, by themselves, establish causal effects (Zawadzki et al., 2023). Accordingly, the present results should be interpreted as hypothesis-generating statistical associations rather than evidence of a causal mechanism. Genome sequencing, repeat-content analysis, gene-copy-number assessment, transcript profiling, and controlled environmental experiments will be required to determine whether genome structure is functionally related to saponin accumulation.

The PLS-PM suggested that temperature was positively associated with the plant-growth construct, whereas the plant-growth construct was negatively associated with the saponin-quality construct (**Figure 10**). Importantly, the negative path coefficient of −0.381 represented the association between plant growth and saponin quality rather than a direct association between temperature and saponin accumulation. The direct temperature-to-saponin path was weak and statistically non-significant. These results may indicate a potential trade-off between vegetative growth and the allocation of resources to secondary metabolism; however, the present observational data do not provide sufficient evidence to demonstrate such a physiological mechanism. The R² values of the endogenous constructs ranged from 0.080 to 0.222, indicating that the model explained only a limited proportion of the variation in karyotypic characteristics, genome size, plant growth, and saponin profiles. The global GoF value was 0.2428; however, this index was treated only as a descriptive statistic because the conventional GoF criterion cannot reliably distinguish valid from invalid PLS path models (Henseler and Sarstedt, 2013). A substantial proportion of the observed variation therefore remained unexplained. Soil nutrient availability, light or shading conditions, cultivation history, plant developmental stage, and rhizosphere microorganisms may contribute to variation in the growth and secondary metabolism of medicinal plants. In P. polyphylla, changes in soil nutrients, enzyme activities, and microbial-community composition have been associated with substantial differences in saponin accumulation (Liu et al., 2024). Light conditions can also modify biomass production and steroidal-saponin accumulation in P. polyphylla, indicating that variation in canopy openness or cultivation shade may represent an important unmeasured factor (Li et al., 2023).

The present path model should be interpreted cautiously. Altitude, temperature, and precipitation were population-level source-site variables, whereas three biological measurements from each population were entered separately into the current model. Measurements from the same population were therefore not fully independent, which may have inflated the effective sample size. This type of non-independence is generally regarded as pseudoreplication and may lead to inappropriate estimation of sampling error and statistical significance (Hulbert and Hulbert, 1984). Moreover, the significance of the current paths was assessed using internal linear-model estimates rather than bootstrap confidence intervals. Predictive performance was not evaluated using a cross-validated out-of-sample prediction procedure. Although the Stone–Geisser Q² obtained through blindfolding is commonly reported as an indicator of predictive relevance, recent methodological studies have emphasized that it should not be interpreted as direct evidence of out-of-sample predictive performance. Procedures such as PLSpredict, which compare cross-validated prediction errors with those of benchmark models, provide a more appropriate assessment of the model’s predictive capability (Shmueli et al., 2016; Shmueli et al., 2019).

Therefore, the present study should be regarded as generating biologically meaningful hypotheses rather than demonstrating causal mechanisms linking genome size, environmental variables, and saponin accumulation. Experimental validation under controlled conditions will be necessary to test these hypotheses.

Collectively, these findings provide a comprehensive view of the ecological–genetic coordination shaping phenotypic stability and saponin diversity in cultivated *P. polyphylla* populations. The integration of cytogenetic, molecular, and metabolic traits establishes a multidimensional framework for germplasm identification, breeding, and conservation of this endangered medicinal species.

## 5 Conclusion

This study integrated morphological, molecular, and biochemical analyses to evaluate 28 cultivated populations of *Paris polyphylla* from Yunnan. Phenological divergence and trait stability varied markedly among populations, with H2 and H8 showing distinct developmental rhythms. Among the representative population sequences examined, ITS exhibited greater haplotype richness and sequence variation than *trnL–trnF*. However, the use of one consensus sequence per population prevented assessment of within-population variation and conventional DNA barcode gaps. Flow cytometry and karyotype analyses confirmed diploidy, while genome size variation indicated evolutionary differentiation. Saponin composition showed strong inter-population variability and was closely associated with agronomic traits, chromosome structure, and genome size. Exploratory path modeling indicated associations among source-site climatic variables, plant growth, genome size, karyotypic characteristics, and saponin profiles. However, the relatively low R² values and the observational structure of the data indicate that these relationships should not be interpreted as direct causal effects. Overall, the findings highlight the interplay of genetic, morphological, and environmental factors shaping growth and phytochemical diversity in *P. polyphylla*, providing a scientific basis for germplasm conservation and cultivar improvement.

## Data Availability

The ITS sequences generated in this study have been deposited in GenBank under accession numbers PZ678994–PZ679021. The trnL–trnFsequences have also been submitted to GenBank under accession number PZ714909-PZ714936.

## Supplementary material

Supplementary Table 1 Statistical comparisons among 28 cultivated populations.

Supplementary Figure 1 Representative HPLC chromatograms of the mixed reference standards and a representative cultivated-population sample. **(A)** Chromatogram of the mixed reference standards containing polyphyllin VII, polyphyllin D, polyphyllin H, polyphyllin VI, polyphyllin II, and polyphyllin I. **(B)** Representative HPLC chromatogram of cultivated population H7. Polyphyllin VII, polyphyllin D, polyphyllin H, polyphyllin II, and polyphyllin I were detected, whereas polyphyllin VI was not detected under the current analytical conditions.

Supplementary Figure 2 Multiple sequence alignment of the ITS sequences obtained from the 28 cultivated populations of Paris polyphylla. Dots indicate nucleotides identical to the reference sequence, nucleotide letters indicate substitution polymorphisms, and hyphens represent alignment gaps corresponding to putative insertion/deletion events.

Supplementary Figure 3 Multiple sequence alignment of the trnL–trnF sequences obtained from the 28 cultivated populations of Paris polyphylla. Dots indicate nucleotides identical to the reference sequence, nucleotide letters indicate substitution polymorphisms, and hyphens represent alignment gaps corresponding to putative insertion/deletion events.
