# Supplementary material for: Multi-dimensional identification and quality evaluation of Paris polyphylla cultivated populations using morphological, molecular, cytological and metabolic traits
Source: Front Plant Sci. 2026 Aug 13;17:1878411. doi: 10.3389/fpls.2026.1878411 (PMC13518599; doi:10.3389/fpls.2026.1878411)
Supplement: Supplementary file 1 [file DataSheet1.docx]

**Supplementary materials**

**Table S1. Statistical comparisons among 28 cultivated populations**

| Variable | Replicates per population (n) | Statistical test | Degrees of freedom | Statistic | F/H value | P-value | Significance | Post-hoc multiple comparison |
| --- | --- | --- | --- | --- | --- | --- | --- | --- |
| Number of leaves | 5 | One-way ANOVA | 27, 112 | F | 3.502 | 1.68e-06 | *** | Tukey's HSD test |
| Number of sepals | 5 | One-way ANOVA | 27, 112 | F | 4.137 | 5.47e-08 | *** | Tukey's HSD test |
| Stem diameter | 5 | One-way ANOVA | 27, 112 | F | 3.961 | 1.40e-07 | *** | Tukey's HSD test |
| Leaf length | 5 | One-way ANOVA | 27, 112 | F | 13.967 | 9.89e-25 | *** | Tukey's HSD test |
| Number of stamens | 5 | One-way ANOVA | 27, 112 | F | 1.321 | 0.158 | ns | Not performed (overall test not significant) |
| Leaf width | 5 | One-way ANOVA | 27, 112 | F | 7.544 | 5.05e-15 | *** | Tukey's HSD test |
| Petiole length | 5 | One-way ANOVA | 27, 112 | F | 4.757 | 2.16e-09 | *** | Tukey's HSD test |
| Pedicel length | 5 | One-way ANOVA | 27, 112 | F | 15.894 | 4.85e-27 | *** | Tukey's HSD test |
| Plant height | 5 | One-way ANOVA | 27, 112 | F | 4.544 | 6.46e-09 | *** | Tukey's HSD test |
| Crown width | 5 | One-way ANOVA | 27, 112 | F | 14.412 | 2.77e-25 | *** | Tukey's HSD test |
| Polyphyllin I | 3 | One-way ANOVA | 27, 56 | F | 18.483 | 3.56e-19 | *** | Tukey's HSD test |
| Polyphyllin II | 3 | One-way ANOVA | 27, 56 | F | 106.833 | 5.40e-39 | *** | Tukey's HSD test |
| Polyphyllin H | 3 | One-way ANOVA | 27, 56 | F | 775.706 | 8.18e-63 | *** | Tukey's HSD test |
| Polyphyllin VII | 3 | One-way ANOVA | 27, 56 | F | 294.771 | 4.01e-51 | *** | Tukey's HSD test |
| Genome size | 3 | One-way ANOVA | 27, 56 | F | 102.760 | 1.56e-38 | *** | Tukey's HSD test |
| Polyphyllin D | 3 | Kruskal–Wallis test | 27 | H | 82.962 | 1.33e-07 | *** | Dunn's test with Benjamini–Hochberg adjustment |

**Figure S1**. Representative HPLC chromatograms of the mixed reference standards and a representative cultivated-population sample.(A) Chromatogram of the mixed reference standards containing polyphyllin VII, polyphyllin D, polyphyllin H, polyphyllin VI, polyphyllin II, and polyphyllin I.(B) Representative HPLC chromatogram of cultivated population H7. Polyphyllin VII, polyphyllin D, polyphyllin H, polyphyllin II, and polyphyllin I were detected, whereas polyphyllin VI was not detected under the current analytical conditions.

**Figure S2**. Multiple sequence alignment of the ITS sequences obtained from the 28 cultivated populations of *Paris polyphylla*. Dots indicate nucleotides identical to the reference sequence, nucleotide letters indicate substitution polymorphisms, and hyphens represent alignment gaps corresponding to putative insertion/deletion events.

**Figure S3**. Multiple sequence alignment of the trnL–trnF sequences obtained from the 28 cultivated populations of Paris polyphylla. Dots indicate nucleotides identical to the reference sequence, nucleotide letters indicate substitution polymorphisms, and hyphens represent alignment gaps corresponding to putative insertion/deletion events.
